# Specialized nursing terminology in pre-exposure prophylaxis: alexical analysis

**DOI:** 10.1590/1980-220X-REEUSP-2026-0166en

**Published:** 2026-07-24

**Authors:** José Leonildo Fernandes de Queiroz, Daniela Dias Quirino, Semíramis Mesquita Ciríaco da Silva, Adelson de Oliveira da Cruz, Isaque Augusto Rosendo Costa, Richardson Augusto Rosendo da Silva

**Affiliations:** 1 Universidade Federal do Rio Grande do Norte, Departamento de Enfermagem, Natal, RN, Brazil.; 2 Universidade Federal da Paraíba, Departamento de Enfermagem, João Pessoa, PB, Brazil.; 3 Universidade Federal do Rio Grande do Norte, Departamento de Psicologia, Natal, RN, Brazil.

**Keywords:** Nursing Care, Disease Prevention, Sexual Health, HIV, Pre-Exposure Prophylaxis.

## Abstract

**Objective:**

To analyze the lexicon of terms related to nursing care directed at users of pre-exposure prophylaxis (PrEP) for HIV, based on the scientific literature.

**Method:**

A scoping review conducted according to PRISMA-ScR guidelines. Studies were identified in multidisciplinary and health-specific databases, national and international repositories of grey literature, and official documents from the Ministry of Health, with no restrictions on language or year. The studies were submitted to lexical analysis using the IRaMuTeQ software, and the findings were interpreted in light of Roy’s Adaptation Model, which guided the organization of lexical classes according to the physiological, self-concept, role function, and interdependence modes, enabling a multidimensional understanding of the phenomenon.

**Results:**

A total of 24,245 records were identified, of which 18 comprised the final corpus, totaling 1,770 text segments. Four lexical classes emerged: Implementation of HIV PrEP, Professional Care, Perception of Vulnerability, and Decision-Making.

**Conclusion:**

A representative vocabulary of nursing care in PrEP was evidenced, highlighting the contribution of Roy’s Theory to the interpretation of findings and to the integrated understanding of the dimensions of nursing care.

## INTRODUCTION

The human immunodeficiency virus (HIV) epidemic remains a global public health challenge, requiring innovative prevention strategies. In this context, pre-exposure prophylaxis (PrEP), based on the use of antiretroviral drugs, has been consolidated as an effective tool to reduce virus transmission and expand protection among vulnerable populations^(1)^.

The Nursing Process (NP) is essential to systematize care and sustain the professional practice of nurses^(2)^. To this end, it is necessary to continuously qualify practice, particularly through the use of Standardized Language Systems (SLS), which enhance multidisciplinary communication and ensure continuity of health actions. Among SLS, the International Classification for Nursing Practice (ICNP®) stands out for its comprehensiveness and ability to support accurate clinical records^(3,4)^.

However, gaps persist in clinical practice regarding the use and documentation of terms related to nursing care in the context of HIV PrEP. This weakness compromises the systematization of actions, language standardization, and the effectiveness of communication among health professionals^(5)^.

Lexical analysis of the scientific literature emerges as a promising strategy to identify the most recurrent concepts on the subject, revealing beliefs, practices, and dimensions of care through nursing’s academic discourse. Lexicography provides a theoretical and methodological foundation to understand language in use, enabling not only the identification of relevant terms but also the organization of meaning clusters into thematic classes representative of everyday practice^(6)^.

In nursing, lexical analysis contributes to the understanding of complex phenomena^(7)^, such as care for PrEP users. It supports the construction of more consistent ICNP® terminological subsets, strengthens the classification system, expands language standardization, and enhances clinical records, thereby justifying the present study.

The relevance of this study lies not only in the innovation of its approach but also in its capacity to inform health policies and qualify professional practices, fostering multidisciplinary communication and precise clinical documentation. This production contributes to consolidating the role of nursing in HIV prevention and PrEP implementation, with direct impact on care quality and user safety.

Given this, the following question arises: how are the terms related to nursing care for HIV PrEP users lexically organized in the scientific literature? Thus, this study aims to analyze the lexicon of terms related to nursing care directed at HIV PrEP users, based on the scientific literature.

## METHOD

### Study Design

This is a scoping review, guided by a research protocol previously registered in the Open Science Framework (https://doi.org/10.17605/OSF.IO/N3PMX). The study was conducted in accordance with the recommendations of the Joanna Briggs Institute Reviewer’s Manual and the PRISMA-ScR guidelines^(8)^.

The guiding question was constructed using the PCC strategy, defined as: P (Population) HIV PrEP users; C (Concept) Terms related to nursing care; and C (Context) Scientific literature on PrEP and nursing care. Thus, the following question was established: Which terms related to nursing care directed at HIV PrEP users are identified and described in the scientific literature?

### Study Setting

Searches were conducted in electronic databases and grey literature repositories, using the CAPES Journals Portal via the Federal Academic Community (CAFe).

### Population and Selection Criteria

Articles aligned with the research objective, published in national and international indexed databases, with no time or language restrictions, were included, as well as grey literature (theses, dissertations, and technical documents). Exclusion criteria comprised studies without complete results or only preliminary notes.

### Data Collection

Studies were identified in multidisciplinary electronic databases and grey literature sources: Scopus; Web of Science; PubMed®; Cumulative Index to Nursing and Allied Health Literature (CINAHL); Cochrane Library; Latin American and Caribbean Literature in Health Sciences (LiLaCS); SciELO; Embase®; Education Resources Information Center (ERIC); Google Scholar; DART-Europe; E-Theses Portal; Electronic Theses Online Service (EthOS); Repositório Científico de Acesso Aberto de Portugal (RCAAP); Global ETD Search; Open Access Theses and Dissertations (OATD); Theses Canada; Portal de Tesis Latinoamericanas; WorldCat Dissertations and Theses (OCLC); CAPES Theses and Dissertations Catalog; Brazilian Digital Library of Theses and Dissertations (BDTD); World Wide Science; Brazilian Ministry of Health protocols and technical notes; opinions from the Federal Nursing Council and Regional Nursing Councils; and manual searches of reference lists from included studies to retrieve additional evidence.

Searches were conducted in November 2025 and structured with indexed descriptors (MeSH/DeCS) and free terms, combined with Boolean operators AND and OR, adapted to each database. The main search keys included: Nursing, Pre-Exposure Prophylaxis, Anti-HIV Agents, Disease Prevention, Sexual Health, Nurse’s Role, Practice Patterns, Nurses. Accordingly, the following search combinations were performed: ((“Nursing” OR “Nurse’s Role” OR “Practice Patterns, Nurses’”) AND (“Pre-Exposure Prophylaxis” OR “Disease Prevention” OR “Anti-HIV Agents”) AND (“Sexual Health”)).

Selection was performed in three stages: screening, full-text reading, and consensus. The Rayyan - Intelligent Systematic Review software (https://rayyan.ai/) was used. Two independent researchers analyzed titles and abstracts; eligible studies were read in full. Divergences were resolved by a third researcher, and duplicates were considered only once.

Data extraction was performed using a structured instrument in Microsoft Excel 2019, conducted by two researchers, with disagreements resolved by consensus. The instrument included study identification (country, year, type of publication, study type, and level of evidence).

### Data Analysis and Treatment

Studies were classified according to the Oxford Centre for Evidence-Based Medicine (OCEBM) levels of evidence and the JBI classification (levels 1 to 4). Evidence ranged from level 1 (robust syntheses) to level 4 (descriptive studies and experience reports).

The textual corpus, composed of abstracts, results, and final considerations of the selected studies, was submitted to IRaMuTeQ software. This program applied statistical techniques of lexical analysis, such as Descending Hierarchical Classification (DHC) and Correspondence Factor Analysis (CFA), which segment text into context units and group terms by semantic similarity and frequency of occurrence. DHC enabled the identification of homogeneous and semantically coherent lexical classes, while CFA highlighted the spatial and relational distribution of these classes, facilitating visualization of connections among meaning clusters. This process ensured methodological rigor and supported interpretation of findings in light of Roy’s Adaptation Model, which guided the organization of classes into physiological, subjective, professional, and relational dimensions of nursing care.

Results were presented through the PRISMA-ScR flowchart (identification, screening, eligibility, and inclusion), tables with methodological characteristics and main findings, tables summarizing levels of evidence and search strategies, and IRaMuTeQ figures (DHC dendrogram and CFA factorial map).

The choice of lexical analysis using IRaMuTeQ was justified by its relevance in identifying meaning clusters and language usage patterns in scientific texts. In the field of ICNP® terminology development, this approach represents an initial stage of mapping and organizing terms, providing support for the elaboration of more consistent terminological subsets. It is therefore a complementary strategy preceding clinical validation processes, contributing to strengthening language standardization and the practical applicability of ICNP® in nursing records.

Furthermore, the choice of Callista Roy’s Adaptation Model as the analytical framework was based on its ability to interpret complex nursing care phenomena through four adaptive modes: physiological, self-concept, role function, and interdependence. This theoretical structure proved suitable for the objective of terminological analysis, as it allowed lexical findings to be organized into dimensions reflecting clinical, biological, subjective, relational, and organizational aspects of care^(9)^.

### Ethical Aspects

As this study is a literature review based on publicly accessible secondary data, submission to a Research Ethics Committee was not required. Nevertheless, ethical principles related to respect for copyright and proper citation of sources were observed.

## RESULTS

**Figure 1:**
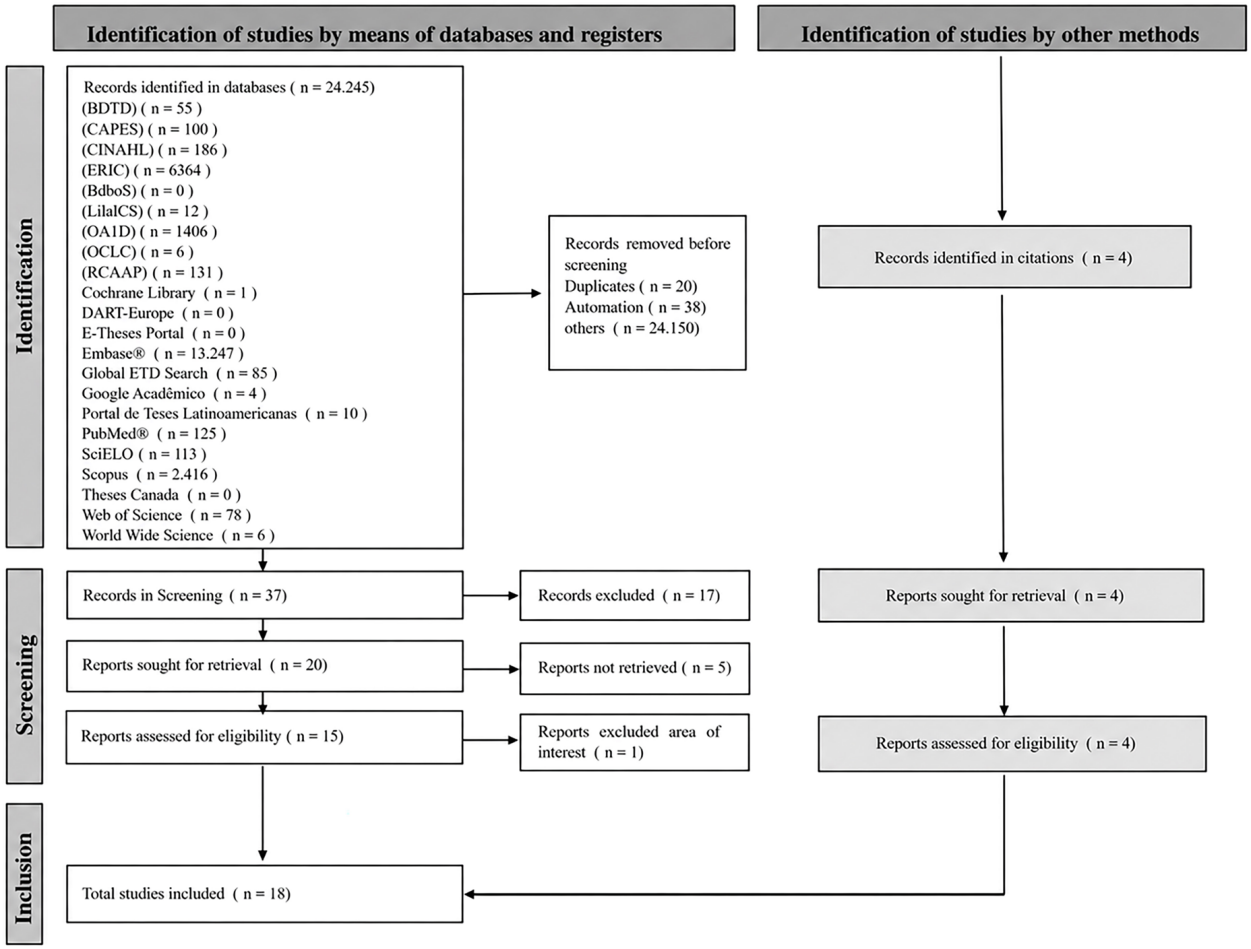
Flowchart for Scoping Review (adapted from PRISMA). Natal, RN, Brazil, 2026

The review process began with the identification of 24,247 records across different databases and through manual searches. During the screening stage, 24,223 records were excluded for not meeting eligibility criteria, duplication, irretrievability, thematic irrelevance, or failure to address the guiding question. In parallel, four reports/manuals analyzed through complementary methods were all retained. At the end of the selection process, 18 studies were included in the final sample of this review (Figure 1).

The final textual corpus of the study consisted of 18 documents, presented in Chart 1, which were processed and segmented into 1,770 text segments (TSs). Of these, 1,566 (88.47%) were classified through lexical analysis. After data analysis, the results were organized and presented in the table. To facilitate understanding and visualization of the findings, the studies were identified with the letter “E” (study), followed by sequential Arabic numerals (1, 2, 3, …18), resulting in the codifications E1, E2, E3, … E18 (Chart 1).

**Chart 1:** Characterization of the studies selected for the research – Natal, RN, Brazil, 2026.

Study	Country (Year)	Type of publication / Type of study / Level of evidence
E1^(6)^	Australia (2018)	Scientific article / Clinical study / 3
E2^(7)^	Canada (2018)	Scientific article / Correlational observational study / 4
E3^(8)^	United States (2019)	Scientific article / Methodological study / 4
E4^(9)^	United States (2019)	Scientific article / Correlational observational study / 4
E5^(10)^	Canada (2020)	Scientific article / Observational study / 4
E6^(11)^	Canada (2020)	Scientific article / Retrospective observational study / 4
E7^(12)^	Canada (2020)	Scientific article / Longitudinal observational study / 4
E8^(13)^	Brazil (2020)	Technical report / Institutional document / NE^*^
E9^(14)^	Canada (2021)	Scientific article / Observational study / 4
E10^(15)^	Canada (2022)	Scientific article / Qualitative study / 5
E11^(16)^	United States (2022)	Scientific article / Clinical trial protocol / 3
E12^(17)^	Brazil (2022)	Guideline/Protocol / Evidence synthesis / NE^*^
E13^(18)^	Canada (2023)	Scientific article / Qualitative study / 4
E14^(19)^	Canada (2023)	Scientific article / Observational study / 4
E15^(20)^	Brazil (2023)	Technical note / Institutional document / NE^*^
E16^(21)^	Brazil (2024)	Technical note / Institutional document / NE^*^
E17^(22)^	United States (2025)	Scientific article / Qualitative study / 4
E18^(23)^	Australia (2025)	Scientific article / Observational study / 3

*NE (Not eligible for OCEBM classification).

**Figure 2:**
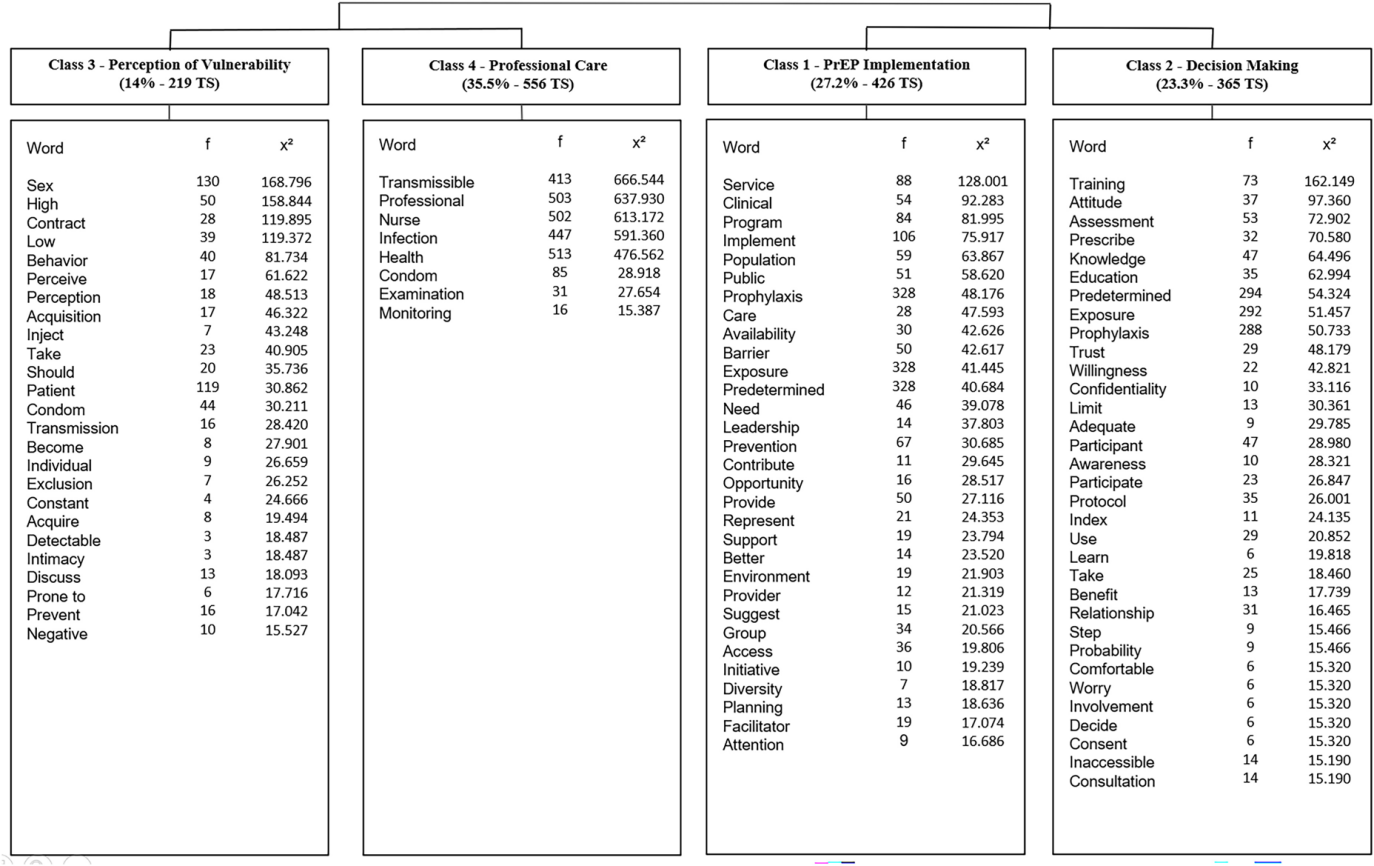
Diagram of the classes comprising the dendrogram of the textual corpus related to the manuscripts in the sample. Natal, RN, Brazil, 2026

The analysis conducted using IRaMuTeQ software enabled the classification of segments from 18 texts into four distinct thematic classes. To ensure terminological uniformity, the classes were named in the singular and standardized as follows: Class 1 – Implementation of PrEP (27.2%); Class 2 – Decision-Making (23.3%); Class 3 – Perception of Vulnerability (14%); Class 4 – Professional Care (35.5%), as presented in Figure 2.

The lexical classes identified do not appear in isolation but are articulated as interdependent stages of the care process related to PrEP. This trajectory begins with the implementation of PrEP in health services (Class 1) and the decision-making process of users (Class 2), which express organizational, programmatic, and cognitive dimensions of care. Subsequently, classes related to the perception of vulnerability (Class 3) and professional care (Class 4) emerge, incorporating subjective, relational, and care-related aspects, including the role of health professionals, particularly nursing, in prevention and follow-up.

The relationship among the classes should be analyzed from left to right, according to the logic of Descending Hierarchical Classification. In this study, the corpus was subjected to a single division, resulting in four subcorpora corresponding to Class 3 (Perception of Vulnerability), Class 4 (Professional Care), Class 1 (Implementation of PrEP), and Class 2 (Decision-Making). This configuration indicates that the classes emerged simultaneously from the same segmentation process, without successive subdivisions of the corpus. The interruption of classification occurred due to the stability achieved, evidenced by the formation of sets of text segments with internally homogeneous and semantically coherent vocabulary, allowing for integrated analysis of the classes as complementary dimensions of the phenomenon under investigation (Figure 2).

**Figure 3:**
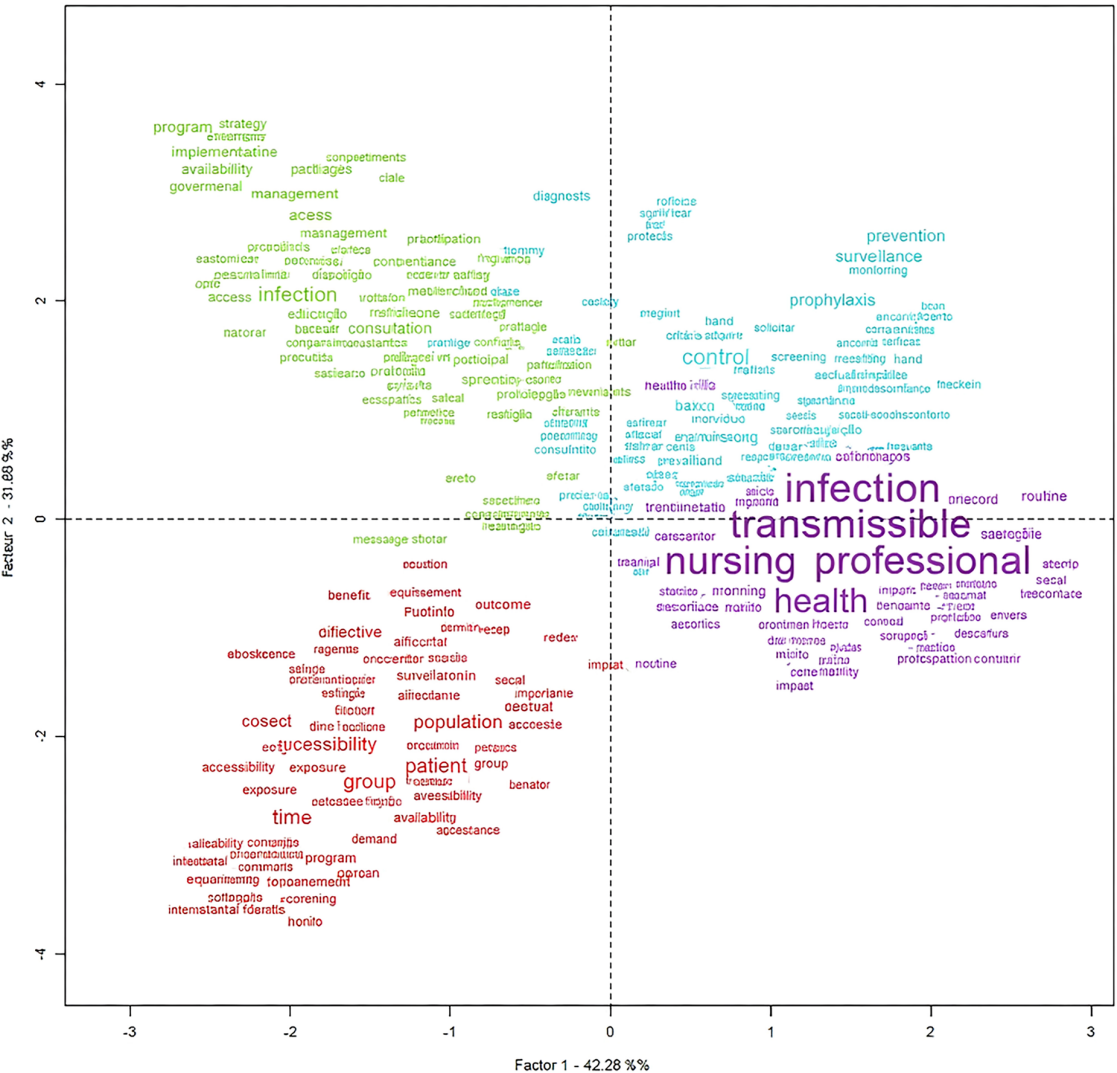
Correspondence factor analysis of the most frequent words in each lexical class, based on hierarchical classification. Natal, RN, Brazil, 2026.

The Correspondence Factor Analysis (CFA) mapped the spatial distribution of these classes (Figure 3). The identified classes are directly articulated with health and nursing care practices in the context of HIV prevention and PrEP implementation, as they represent essential dimensions of the care process.

The lexical findings related to PrEP implementation and decision-making (Classes 1 and 2) impact service organization, resource availability, and access conditions, while also being linked to educational, informational, and ethical processes that support conscious choices and adherence to preventive strategies. In turn, the classes concerning perception of vulnerability and professional care (Classes 3 and 4) reinforce the importance of understanding individual vulnerability, preventive behavior, and the bond established between users and health professionals.

The Correspondence Factor Analysis (CFA) highlighted the semantic and lexical organization of the most frequent terms in the corpus, allowing visualization of the relational structure among the identified classes. As illustrated in the factorial plane, Class 2 – Decision-Making (upper left quadrant) concentrates terms associated with training, knowledge, education, evaluation, confidence, consent, and attitude, indicating a strong articulation between cognitive, informational, and decision-making processes. The position of this class suggests the centrality of formative aspects and individual autonomy in the analyzed context.

In the lower left quadrant, Class 1 – Implementation of PrEP is characterized by terms such as service, program, implement, access, availability, population, barrier, and prevention. The spatial proximity between Classes 1 and 2 indicates a high semantic correlation, revealing that decision-making processes are intrinsically related to organizational and programmatic conditions of health services responsible for providing PrEP.

In the upper right quadrant, Class 3 – Perception of Vulnerability stands out, composed of terms such as sex, behavior, exposure, condom, and transmission. This class expresses the subjective and behavioral dimension of care, related to individual interpretation of vulnerability and preventive practices. Its position in the factorial plane indicates relative conceptual autonomy, although it maintains interfaces with other domains of care.

Finally, in the lower right quadrant, Class 4 – Professional Care is strongly associated with terms such as nurse, professional, health, infection, transmissible, monitoring, and examination. The relative isolation of this class in the factorial plane highlights the specificity of the lexical field linked to clinical practice and technical-professional knowledge, indicating that, although related to other classes, this dimension is structured around its own conceptual core.

Taken together, the distribution of classes in the factorial plane reveals the complexity of the discourse analyzed, by showing how organizational, decision-making, subjective, and technical-care dimensions are articulated in the context of HIV prevention and PrEP implementation. CFA thus enables an integrated reading of the association patterns between terms and classes, reinforcing the multidimensional nature of health care and nursing practice.

Chart 2 terms extracted from the corpus organized according to the four adaptive modes of Roy’s Adaptation Model, evidencing the multidimensional nature of the phenomenon analyzed in the context of HIV prevention and PrEP implementation.

**Chart 2:** Terms related to care in the context of HIV PrEP in light of Callista Roy’s Adaptation Model – Natal, RN, Brazil, 2026.

Adaptive modes	Related terms
Physiological mode	Transmissible; Infection; Health; Condom; Examination; Monitoring; Prophylaxis; Exposure; High; Contract; Acquisition; Acquire; Inject; Take; Transmission; Detectable; Negative; Prevent; Probability; Index.
Self-concept mode	Attitude; Confidence; Disposition; Awareness; Perception; Perceive; Comfortable; Worry; Benefit; Decide; Duty; Become.
Role function mode	Professional; Nurse; Training; Evaluation; Prescribe; Knowledge; Education; Protocol; Use; Learn; Stage; Consultation; Service; Clinical; Program; Implement; Leadership; Provider; Planning; Initiative.
Interdependence mode	Participant; Participate; Relationship; Involvement; Consent; Confidentiality; Patient; Individual; Population; Public; Group; Diversity; Care; Access; Availability; Barrier; Need; Support; Provide; Opportunity; Environment; Attention; Suggest; Represent; Exclusion; Inaccessible; Discuss; Intimacy.

This is an open-access article distributed under the terms of the Creative Commons Attribution License.

The physiological mode encompasses terms related to infection, transmission, exposure, prophylaxis, examination, and monitoring, expressing the biological and clinical aspects of care. The self-concept mode aggregates terms linked to perception, attitude, confidence, disposition, and decision-making, reflecting the subjective and psychological dimension of the adaptive process. The role function mode concentrates words associated with professional performance, such as nurse, training, evaluation, protocol, prescription, service, and implementation, highlighting the technical, educational, and organizational role of nursing. Finally, the interdependence mode includes terms related to participation, bonding, confidentiality, access, support, environment, and relationship, evidencing the importance of social and institutional interactions in care.

## DISCUSSION

The temporal analysis of publications between 2018 and 2025 shows a progressive concentration of studies beginning in 2018, demonstrating the growing scientific interest in PrEP. This configuration follows consolidated trends in the international literature, which highlight the predominance of research centers in the Global North in PrEP-related scientific production, while simultaneously evidencing the strengthening participation of Latin American countries, particularly Brazil, in analyses of implementation within public health systems.

Lexical analysis revealed the discursive construction of the HIV PrEP phenomenon, based on perceptions, practices, and decisions related to health care. The grouping performed through IRaMuTeQ allowed the identification of meaning clusters that express different conceptual axes involved in implementation, access, and decision-making regarding PrEP. Although the organization of classes results from the statistical processing of the software, clinical inference was grounded in the researchers’ interpretation^(3)^.

The lexical classes were standardized according to semantic axes representing: perception of vulnerability, professional care, PrEP implementation, and decision-making. Each class gathers interrelated terms that, collectively, deepen the understanding of barriers, strategies, and processes permeating HIV prevention in health services, as evidenced in the dendrogram and Correspondence Factor Analysis.

CFA demonstrated that, in Class 1, terms highlight organizational and programmatic challenges such as access, availability, and barriers. This underscores the need for clear protocols to ensure PrEP provision in different care contexts, the importance of preparing nurses to address structural and service management issues, and the implementation of public policies that guarantee resources and adequate care flows, expanding coverage and equity in access^(10,11,12)^.

Class 2 concentrated terms linked to knowledge, confidence, consent, and attitude, reflecting cognitive and ethical processes. These findings point to the need for nurses to adopt educational strategies that promote conscious choices and adherence to PrEP, to understand factors influencing users’ decisions regarding its use, and to develop awareness campaigns that reduce informational barriers^(13,14,15)^.

In Class 3, terms related to vulnerability highlighted the demand for user-centered practices that consider individual risk perception and foster trust bonds. The importance of training nurses with competencies in communication and culturally sensitive approaches is emphasized. By understanding users’ perceptions of vulnerability, nurses can guide prevention strategies more targeted to specific groups, thereby increasing the effectiveness of health policies^(16,17)^.

Class 4 gathered terms that evidenced the technical-care core of nursing practice. This finding reinforces the role of nurses as key actors in PrEP delivery, from prescription to clinical follow-up. It also highlights the need for continuous training in protocols and evidence-based practices, as well as terminological standardization as a tool to strengthen care indicators, assess quality of care, and support strategic decision-making^(18,19)^.

Regarding the connection among classes, proximity was observed between Class 1 and Class 4, evidenced by the concentration of terms related to services, programs, clinical practice, and professional activities. This association indicates that PrEP effectiveness is directly linked to the organizational capacity of health services, action planning, and the qualified performance of professionals, especially nurses, in leading HIV preventive care^(20,21,22,23)^. The connection between Class 3 and Class 2 reveals that the proximity of terms related to exposure, behavior, and prevention with words associated with attitude, confidence, knowledge, and decision-making^(24,25)^, demonstrates that the choice to use PrEP goes beyond the technical dimension, involving subjective, cognitive, and emotional processes of individuals.

The use of Roy’s Adaptation Model as a theoretical framework made it possible to highlight how each mode translates specific dimensions of nursing care related to HIV PrEP, enabling the understanding of clinical and biological aspects as well as subjective, professional, and relational components of the care process. Thus, the association between a nursing theory and lexical analysis expanded the practical applicability of the results, offering support for nursing care, education, and management by aligning technical interventions with the human and social needs of users.

Terms related to the physiological mode, such as infection, transmission, examination, monitoring, and prophylaxis, highlight the biological and clinical dimension of nursing care in PrEP. In practice, this implies more precise protocols for screening and clinical follow-up, ensuring that users receive continuous and safe monitoring^(26,27)^. In education, it reinforces the need to train nurses to interpret laboratory tests and provide guidance on preventive measures. In management, the standardization of these records strengthens epidemiological indicators and allows better resource planning for HIV prevention.

The self-concept mode, represented by terms such as attitude, confidence, perception, and decision-making, reveals the subjective and psychological dimension of care. In practice, nurses must develop communication strategies that promote users’ confidence and autonomy in choosing PrEP. In education, the findings point to the importance of including content on counseling, empowerment, and decision-making in nursing curricula. In management, understanding these subjective aspects helps formulate policies that value adherence and reduce emotional or cultural barriers to PrEP use.

Terms linked to the role function mode, such as nurse, training, prescription, protocol, and implementation, highlight the professional and organizational dimension of nursing. In practice, this reinforces the nurse’s role as a protagonist in PrEP implementation, from prescription to clinical follow-up^(28)^. In education, it emphasizes the need to prepare professionals to lead prevention programs, with a focus on protocols and evidence-based practices. In management, terminological clarity strengthens the construction of care flows and the definition of responsibilities, ensuring greater efficiency in health services.

The interdependence mode, expressed by terms such as bonding, consent, confidentiality, access, and support, highlights the relational and social dimension of care. In practice, this translates into approaches that value therapeutic bonds and confidentiality, which are fundamental for vulnerable populations^(29,30)^. In education, the results reinforce the need to train nurses with competencies in communication, cultural diversity, and ethics. In management, the standardization of these aspects supports inclusive policies, expands access, and strengthens support networks, ensuring that PrEP is implemented equitably and centered on users’ needs.

This integration makes it possible to align technical interventions with the social and human dimensions of care, offering support for nurses’ clinical reasoning and for the development of more effective, equitable, and user-centered interventions for populations using or potentially eligible for PrEP.

The limitation of this study concerns the exclusive use of IRaMuTeQ software, which, although robust, depends on the consistency of the textual corpus and the researchers’ interpretation for term categorization. This characteristic may restrict the generalization of results, as lexical analysis is conditioned by the quality and context of the texts included.

Despite these limitations, the results of this lexical analysis present direct practical implications for nursing in different dimensions. In practice, the identification of recurrent terms can guide the construction of more standardized clinical recording protocols, favoring the use of ICNP® and ensuring greater precision in care documentation. This strengthens patient safety and ensures continuity of health actions.

In education, the findings indicate priority content for professional training, such as decision-making, perception of vulnerability, and PrEP implementation. These elements can be incorporated into undergraduate curricula and training programs, preparing nurses to deal with complex scenarios of HIV prevention and expanding their clinical and educational competence.

In management, terminological standardization contributes to the qualification of care indicators, monitoring of results, and strengthening of public health policies. This practice allows managers to use more consistent data in the formulation of prevention strategies and resource allocation. Thus, the study offers concrete support to improve clinical practice, academic training, and nursing management, expanding the impact of professional performance in HIV prevention.

## CONCLUSION

This study highlighted the multidimensional nature of nursing care in the context of HIV PrEP, by revealing lexical classes that articulate organizational, subjective, professional, and relational dimensions. The results contribute to strengthening nurses’ clinical reasoning, supporting the standardization of records, and enhancing the safety and quality of care.

Practically, the findings provide guidance for professional practice at different levels: they inform more consistent clinical documentation, support educational processes, and subsidize health management policies. The terminological clarity identified favors multidisciplinary communication and reinforces the strategic role of nursing in HIV prevention.

For future perspectives, it is essential to advance terminological standardization linked to ICNP®, with the development of specific subsets for PrEP and their clinical validation, ensuring greater applicability of terms in care practice. Furthermore, investment in nurse training programs for the consistent use of standardized language is recommended, thereby increasing the effectiveness of prevention strategies and consolidating evidence-based practices.

## Data Availability

The entire dataset supporting the results of this study is available upon request to the corresponding author.
