# Supplementary material for: Specialized nursing terminology in pre-exposure prophylaxis: alexical analysis
Source: Rev Esc Enferm USP. 2026 Jul 24;60:e20260166. doi: 10.1590/1980-220X-REEUSP-2026-0166en (PMC13404302; doi:10.1590/1980-220X-REEUSP-2026-0166en)
Supplement: Translation

# Translation: Terminologia especializada de enfermagem na profilaxia pré-exposição: análise lexical

**DOI:** 10.1590/1980-220X-REEUSP-2026-0166pt

**Authors:** José Leonildo Fernandes de Queiroz, Daniela Dias Quirino, Semíramis Mesquita Ciríaco da Silva, Adelson de Oliveira da Cruz, Isaque Augusto Rosendo Costa, Richardson Augusto Rosendo da Silva

**Affiliations:** 1 Universidade Federal do Rio Grande do Norte, Departamento de Enfermagem, Natal, RN, Brazil.; 2 Universidade Federal da Paraíba, Departamento de Enfermagem, João Pessoa, PB, Brazil.; 3 Universidade Federal do Rio Grande do Norte, Departamento de Psicologia, Natal, RN, Brazil.

**Keywords:** Cuidados de Enfermagem, Prevenção de Doenças, Saúde Sexual, HIV, Profilaxia Pré-Exposição.

## Abstract

**Objetivo:**

Analisar o léxico dos termos referentes ao cuidado de enfermagem direcionado aos usuários da profilaxia pré-exposição (PrEP) para o HIV, com base na literatura científica.

**Método:**

Revisão de escopo conduzida conforme as diretrizes do PRISMA-ScR. A identificação dos estudos ocorreu em bases de dados multidisciplinares e específicas da área da saúde, em repositórios de literatura cinzenta nacionais e internacionais, além de documentos oficiais do Ministério da Saúde, sem restrição de idioma ou ano. Os estudos foram submetidos à análise lexical pelo software IRaMuTeQ, e os achados foram interpretados à luz da Teoria da Adaptação de Roy, que orientou a organização das classes lexicais segundo os modos fisiológico, autoconceito, desempenho de papel e interdependência, permitindo compreender o fenômeno de forma multidimensional.

**Resultados:**

Foram identificados 24.245 registros, dos quais 18 compuseram o corpus final, totalizando 1.770 segmentos de texto. Emergiram quatro classes lexicais: Implementação da PrEP para o HIV, Cuidado Profissional, Percepção de Vulnerabilidade e Tomada de Decisão.

**Conclusão:**

Evidenciou-se um vocabulário representativo do cuidado na PrEP, destacando-se a contribuição da Teoria de Roy para a interpretação dos achados e para a compreensão integrada das dimensões do cuidado de enfermagem.

## INTRODUÇÃO

A epidemia do vírus da imunodeficiência humana (HIV) permanece como um desafio global de saúde pública, exigindo estratégias inovadoras de prevenção. Nesse contexto, a profilaxia pré-exposição (PrEP), baseada no uso de medicamentos antirretrovirais, consolidou-se como uma ferramenta eficaz para reduzir a transmissão do vírus e ampliar a proteção em populações vulneráveis^(1)^.

O Processo de Enfermagem (PE) é essencial para sistematizar o cuidado e sustentar o exercício profissional do enfermeiro^(2)^. Para tanto, torna-se necessário qualificar continuamente a prática, sobretudo por meio do uso de Sistemas de Linguagem Padronizada (SLP), que favorecem a comunicação multiprofissional e a continuidade das ações de saúde. Entre os SLP, destaca-se a Classificação Internacional para a Prática de Enfermagem (CIPE®), reconhecida por sua abrangência e capacidade de subsidiar registros clínicos precisos^(3,4)^.

Contudo, observa-se que, na prática clínica, persistem lacunas no uso e no registro dos termos relacionados ao cuidado de enfermagem no contexto da PrEP para HIV. Essa fragilidade compromete a sistematização das ações, a padronização da linguagem e a efetividade da comunicação entre profissionais de saúde^(5)^.

A análise lexical da literatura científica surge, nesse sentido, como estratégia promissora para identificar os conceitos mais recorrentes sobre o tema, revelando crenças, práticas e dimensões do cuidado à luz dos discursos acadêmicos da enfermagem. A lexicografia fornece base teórica e metodológica para compreender a linguagem em uso, permitindo não apenas a identificação de termos relevantes, mas também a organização de núcleos de sentido em classes temáticas representativas do cotidiano da prática^(6)^.

Na enfermagem, a análise léxica contribui para compreender fenômenos complexos^(7)^, como o cuidado aos usuários da PrEP. Favorece a construção de subconjuntos terminológicos da CIPE^®^ mais consistentes, fortalece o sistema classificatório, amplia a padronização da linguagem e qualifica os registros clínicos, justificando a realização do presente estudo.

A relevância do estudo reside não apenas na inovação da abordagem, mas também em sua capacidade de subsidiar políticas de saúde e práticas profissionais mais qualificadas, favorecendo a comunicação multiprofissional e o registro clínico preciso. Trata-se de uma produção que contribui para consolidar o papel da enfermagem na prevenção do HIV e na implementação da PrEP, com impacto direto na qualidade do cuidado e na segurança dos usuários.

Diante disso, questiona-se: como os termos relacionados ao cuidado de enfermagem aos usuários da PrEP para HIV estão organizados lexicalmente na literatura científica? Assim, este estudo tem como objetivo analisar o léxico dos termos referentes ao cuidado de enfermagem direcionado aos usuários da PrEP para o HIV, com base na literatura científica.

## MÉTODO

### Desenho do Estudo

Trata-se de uma *scoping review*, orientada por protocolo de pesquisa previamente registrado no Open Science Framework (https://doi.org/10.17605/OSF.IO/N3PMX). O estudo foi conduzido conforme as recomendações do *Joanna Briggs Institute Reviewer’s Manual* e as diretrizes PRISMA-ScR^(8)^.

A questão norteadora foi construída pela estratégia PCC, definida como: P (Population) Usuários da PrEP para HIV; C (Concept) Termos relacionados ao cuidado de enfermagem; e C (Context) Literatura científica sobre PrEP e cuidado de enfermagem. Assim, estabeleceu-se a seguinte pergunta: Quais termos relacionados ao cuidado de enfermagem dirigidos aos usuários da PrEP para HIV são identificados e descritos na literatura científica?

### Local do Estudo

As buscas foram realizadas em bases de dados eletrônicas e repositórios de literatura cinzenta, utilizando o Portal de Periódicos da CAPES, por meio da Comunidade Acadêmica Federadal (CAFe).

### População e Critérios de Seleção

Foram incluídos artigos alinhados ao objetivo da pesquisa, publicados em bases indexadas nacionais e internacionais, sem recorte temporal ou limitação de idioma, além de literatura cinzenta (teses, dissertações e documentos técnicos) e como critérios de exclusão, estudos sem resultados completos ou apenas notas prévias.

### Coleta de Dados

A identificação dos estudos ocorreu nas bases de dados eletrônicas multidisciplinares e literatura cinzenta: Scopus; Web of Science; PubMed®; Cumulative Index to Nursing and Allied Health Literature (CINAHL); Cochrane Library; Literatura Latino-Americana e do Caribe em Ciências da Saúde (LiLaCS); SciELO; Embase®; Education Resources Information Center (ERIC); Google Acadêmico; DART-Europe; E-Theses Portal; Electronic Theses Online Service (EthOS); Repositório Científico de Acesso Aberto de Portugal (RCAAP); Global ETD Search; Open Access Theses and Dissertations (OATD); Theses Canada; Portal de Tesis Latinoamericanas; World Cat Dissertations and Theses (OCLC); Catálogo de Teses e Dissertações (CAPES); Biblioteca Digital Brasileira de Teses e Dissertações (BDTD); World Wide Science; Protocolos do Ministério da Saúde do Brasil; Notas Técnicas do Ministério da Saúde do Brasil; Pareceres do Conselho Federal de Enfermagem e dos Conselhos Regionais de Enfermagem do Brasil e busca manual listas de referências dos estudos incluídos, para recuperar evidências não identificadas previamente.

As buscas foram realizadas em novembro de 2025 e estruturadas com descritores indexados (MeSH/DeCS) e termos livres, combinados com os operadores booleanos AND e OR, adaptados a cada base. Principais chaves de busca: *Nursing, Pre-Exposure Prophylaxis, Anti-HIV Agents, Disease Prevention, Sexual Health, Nurse’s Role, Practice Patterns, Nurse’s.* Para tanto foram realizados os seguintes cruzamentos: ((“Nursing” or “Nurse’s Role” or “Practice Patterns, Nurses’”) and (“Pre-Exposure Prophylaxis” or “Disease Prevention” or “Anti-HIV Agents”) and (“Sexual Health”)).

A seleção dos estudos foi realizada em três etapas: triagem, leitura integral e consenso. Para tanto, utilizou-se o software Rayyan - Intelligent Systematic Review (https://rayyan.ai/). Dois pesquisadores, de forma independente, analisaram títulos e resumos; os estudos elegíveis foram lidos na íntegra. Divergências foram resolvidas por um terceiro pesquisador, e as duplicatas foram consideradas apenas uma vez.

A extração dos dados ocorreu por meio de instrumento estruturado no Microsoft Excel 2019, conduzida pelos dois pesquisadores, com divergências solucionadas por consenso. O instrumento contemplou identificação do estudo (país, ano, tipo de publicação, tipo de estudo, e nível de evidência).

### Análise e Tratamento dos Dados

Os estudos foram classificados segundo o nível de evidência do Oxford Centre for Evidence-Based Medicine (OCEBM), segundo a classificação do JBI (níveis 1 a 4). As evidências variaram de nível 1 (sínteses robustas) a nível 4 (estudos descritivos e relatos de experiência).

O corpus textual, constituído a partir de resumos, resultados e considerações finais dos estudos selecionados, foi submetido ao software IRaMuTeQ. Esse programa aplicou técnicas estatísticas de análise lexical, como a Classificação Hierárquica Descendente (CHD) e a Análise Fatorial de Correspondência (AFC), que permitem segmentar o texto em unidades de contexto e agrupar os termos por similaridade semântica e frequência de ocorrência. A CHD possibilitou identificar classes lexicais homogêneas e semanticamente coerentes, enquanto a AFC evidenciou a distribuição espacial e relacional dessas classes, favorecendo a visualização das conexões entre os núcleos de sentido. Esse processo garantiu rigor metodológico e sustentou a interpretação dos achados à luz da Teoria da Adaptação de Roy, que orientou a organização das classes em dimensões fisiológicas, subjetivas, profissionais e relacionais do cuidado de enfermagem.

Os resultados foram apresentados por meio do fluxograma PRISMA-ScR (identificação, triagem, elegibilidade e inclusão), quadro com características metodológicas e principais achados, quadros com síntese do nível de evidência e estratégias de busca, e figuras do IRaMuTeQ (dendrograma da CHD e mapa fatorial da AFC).

A opção pela análise lexical por meio do software IRaMuTeQ foi justificada por sua pertinência na identificação de núcleos de sentido e padrões de uso da linguagem em textos científicos. No campo da construção de terminologias da CIPE®, essa abordagem representa uma etapa inicial de mapeamento e organização dos termos, fornecendo subsídios para a elaboração de subconjuntos terminológicos mais consistentes. Trata-se, portanto, de uma estratégia complementar que antecede processos de validação clínica, contribuindo para fortalecer a padronização da linguagem e a aplicabilidade prática da CIPE® nos registros de enfermagem.

Ademais, a escolha da Teoria da Adaptação de Callista Roy como referencial analítico do estudo, fundamentou-se em sua capacidade de interpretar fenômenos complexos do cuidado de enfermagem a partir de quatro modos adaptativos, fisiológico, autoconceito, desempenho de papel e interdependência. Essa estrutura teórica mostrou-se adequada ao objetivo de análise terminológica, pois^(9)^ permitiu organizar os achados lexicais em dimensões que refletem tanto aspectos clínicos e biológicos quanto subjetivos, relacionais e organizacionais do cuidado.

### Aspectos Éticos

Por se tratar de uma revisão da literatura baseada em dados secundários de acesso público, não houve necessidade de submissão ao Comitê de Ética em Pesquisa. Ressalta-se, contudo, que foram observados os princípios éticos relacionados ao respeito aos direitos autorais e à adequada citação das fontes.

## RESULTADOS

O processo de revisão iniciou-se com a identificação de 24.247 registros em diferentes bases de dados e por busca manual. Na etapa de triagem, 24.223 registros foram excluídos por não atenderem aos critérios de elegibilidade, duplicidade ou irrecuperabilidade, por irrelevância temática ou por não responderem à questão norteadora. Paralelamente, dos quatro relatórios/manuais analisados por métodos complementares, todos foram mantidos. Ao final do processo de seleção, 18 estudos foram incluídos na amostra final desta revisão (Figura 1).

**Figura 1:** Fluxograma para Scoping Review (adaptado do Prisma). Natal, RN, Brasil, 2025.

O corpus textual final da pesquisa foi composto por 18 documentos, apresentados no Quadro 1, os quais foram processados e segmentados em 1770 segmentos de texto (STs), 1566 (88,47%) foram classificados pela análise lexical. Após a análise dos dados, estes foram organizados e apresentados no quadro. Para facilitar a compreensão e a visualização dos resultados, os estudos foram identificados com a letra “E” (estudo), seguida por números arábicos sequenciais (1, 2, 3,...18), originando as codificações E1, E2, E3,... E18 (Quadro 1).

**Quadro 1:** Caracterização dos estudos selecionados para a pesquisa –Natal, RN, Brasil, 2025.

Estudo	País(ano)	Tipo de publicação/ Tipo de estudo / Nível de evidência
E1^(6)^	Austrália (2018)	Artigo científico / Estudo clínico / 3
E2^(7)^	Canadá (2018)	Artigo científico / Estudo observacional correlacional / 4
E3^(8)^	Estados Unidos (2019)	Artigo científico / Estudo metodológico / 4
E4^(9)^	Estados Unidos (2019)	Artigo científico / Estudo observacional correlacional / 4
E5^(10)^	Canadá (2020)	Artigo científico / Estudo observacional / 4
E6^(11)^	Canadá (2020)	Artigo científico / Estudo observacional retrospectivo / 4
E7^(12)^	Canadá (2020)	Artigo científico / Estudo observacional longitudinal / 4
E8^(13)^	Brasil (2020)	Parecer técnico / Documento institucional / NE^*^
E9^(14)^	Canadá (2021)	Artigo científico / Estudo observacional / 4
E10^(15)^	Canadá (2022)	Artigo científico / Estudo qualitativo / 5
E11^(16)^	Estados Unidos (2022)	Artigo científico / Protocolo de ensaio clínico / 3
E12^(17)^	Brasil (2022)	Diretriz/Protocolo / Síntese de evidências / NE^*^
E13^(18)^	Canadá (2023)	Artigo científico / Estudo qualitativo / 4
E14^(19)^	Canadá (2023)	Artigo científico / Estudo observacional / 4
E15^(20)^	Brasil (2023)	Nota técnica / Documento institucional / NE^*^
E16^(21)^	Brasil (2024)	Nota técnica / Documento institucional / NE^*^
E17^(22)^	Estados Unidos (2025)	Artigo científico / Estudo qualitativo / 4
E18^(23)^	Austrália (2025)	Artigo científico / Estudo observacional / 3

*NE (não elegíveis para classificação OCEBM).

A análise conduzida pelo software IRaMuTeQ permitiu a classificação dos segmentos de 18 textos em quatro classes temáticas distintas. Buscando uniformidade terminológica, as classes foram nomeadas no singular e padronizadas da seguinte forma: Classe 1 - Implementação da PrEP (27,2%); Classe 2 - Tomada de decisão (23,3%); Classe 3 - Percepção de Vulnerabilidade (14%); Classe 4 - Cuidado Profissional (35,5%), conforme apresentado na (Figura 2).

**Figura 2:** Diagrama das classes integrantes do dendograma do corpus textual referente aos manuscritos da amostra. Natal, RN, Brasil, 2025.

As classes lexicais identificadas não se apresentam de forma isolada, mas articulam-se como etapas interdependentes do processo de cuidado relacionado à PrEP. Esse percurso inicia-se pela implementação da PrEP nos serviços de saúde (Classe 1) e pela tomada de decisão dos usuários (Classe 2), que expressam dimensões organizacionais, programáticas e cognitivas do cuidado. Em seguida, emergem as classes relacionadas à percepção de Vulnerabilidade (Classe 3) e ao cuidado profissional (Classe 4), que incorporam aspectos subjetivos, relacionais e assistenciais, incluindo o papel do profissional de saúde, especialmente da enfermagem, na prevenção e no acompanhamento.

A relação entre as classes deve ser analisada da esquerda para a direita, conforme a lógica da Classificação Hierárquica Descendente. No presente estudo, o corpus foi submetido a uma única divisão, da qual resultaram quatro subcorpus, correspondentes às Classes 3 (Percepção de Vulnerabilidade), 4 (Cuidado Profissional), 1 (Implementação da PrEP) e 2 (Tomada de Decisão). Essa configuração indica que as classes emergiram simultaneamente a partir do mesmo processo de segmentação, sem subdivisões sucessivas do corpus. A interrupção da classificação ocorreu em razão da estabilidade alcançada, evidenciada pela constituição de conjuntos de segmentos de texto com vocabulário internamente homogêneo e semanticamente coerente, permitindo a análise integrada das classes como dimensões complementares do fenômeno investigado (Figura 2).

A Análise Fatorial de Correspondência (AFC) mapeou a distribuição espacial dessas classes (Figura 3) As classes identificadas articulam-se diretamente com a prática do cuidado em saúde e da enfermagem no contexto da prevenção do HIV e da implementação da PrEP, ao traduzirem dimensões essenciais do processo assistencial.

**Figura 3:** Análise Fatorial de Correspondência das palavras mais frequentes em cada classe lexical, com base em uma classificação hierárquica. Natal, RN, Brasil, 2025.

Os achados lexicais relacionadas à implementação da PrEP e à tomada de decisão (Classes 1 e 2) impactam a organização dos serviços, a disponibilidade de recursos e as condições de acesso, ao mesmo tempo em que se articulam aos processos formativos, informacionais e éticos que sustentam escolhas conscientes e a adesão às estratégias preventivas. Por sua vez, as classes referentes à percepção de vulnerabilidade e ao cuidado profissional (Classes 3 e 4) reforçam a importância da compreensão da vulnerabilidade individual, do comportamento preventivo e do vínculo estabelecido entre usuários e profissionais de saúde.

A Análise Fatorial de Correspondência evidenciou a organização semântica e lexical dos termos mais frequentes no corpus, permitindo visualizar a estrutura relacional entre as classes identificadas. Conforme ilustrado no plano fatorial, a Classe 2 - Tomada de Decisão (representada no quadrante superior esquerdo) concentra termos associados a treinamento, conhecimento, educação, avaliação, confiança, consentimento e atitude, indicando uma forte articulação entre processos cognitivos, informacionais e decisórios. A posição dessa classe sugere centralidade dos aspectos formativos e da autonomia dos sujeitos no contexto analisado.

No quadrante inferior esquerdo, observa-se a Classe 1 - Implementação da PrEP, caracterizada por termos como serviço, programa, implementar, acesso, disponibilidade, população, barreira e prevenção. A proximidade espacial entre as Classes 1 e 2 indica elevada correlação semântica, revelando que os processos decisórios estão intrinsecamente relacionados às condições organizacionais e programáticas dos serviços de saúde responsáveis pela oferta da PrEP.

No quadrante superior direito, destaca-se a Classe 3 - Percepção de Vulnerabilidade, composta por termos como sexo, comportamento, exposição, preservativo e transmissão. Essa classe expressa a dimensão subjetiva e comportamental do cuidado, relacionada à interpretação individual da vulnerabilidade e às práticas de prevenção. Sua posição no plano fatorial indica uma relativa autonomia conceitual, embora mantenha interface com os demais domínios do cuidado.

Por fim, no quadrante inferior direito, concentra-se a Classe 4 - Cuidado Profissional, fortemente associada a termos como enfermeiro, profissional, saúde, infecção, transmissível, monitoramento e exame. O isolamento relativo dessa classe no plano fatorial evidencia a especificidade do campo lexical ligado à prática clínica e ao saber técnico-profissional, indicando que, embora relacionada às demais classes, essa dimensão se estrutura a partir de um núcleo conceitual próprio.

Em conjunto, a distribuição das classes no plano fatorial revela a complexidade do discurso analisado, ao explicitar como dimensões organizacionais, decisórias, subjetivas e técnico-assistenciais se articulam no contexto da prevenção do HIV e da implementação da PrEP. A AFC possibilita, assim, uma leitura integrada dos padrões de associação entre termos e classes, reforçando a natureza multidimensional do cuidado em saúde e da prática da enfermagem.

No Quadro 2, os termos extraídos do corpus foram organizados segundo os quatro modos adaptativos da Teoria da Adaptação de Roy, evidenciando a natureza multidimensional do fenômeno analisado no contexto da prevenção do HIV e da implementação da PrEP.

**Quadro 2:** Termos relacionados ao cuidado no contexto da PrEP ao HIV à luz do referencial teórico da Adaptação de Callista Roy – Natal, RN, Brasil, 2025.

Referencial teórico de adaptação de roy	
Modos adaptativos	Termos relacionados
Modo Fisiológico	Transmissível; Infecção; Saúde; Preservativo; Exame; Monitoramento; Profilaxia; Exposição; Alto; Contrair; Aquisição; Adquirir; Injetar; Tomar; Transmissão; Detectável; Negativo; Prevenir; Probabilidade; Índice.
Modo de Autoconceito	Atitude; Confiança; Disposição; Conscientização; Percepção; Perceber; Confortável; Preocupar; Benefício; Decidir; Dever; Tornar.
Modo de Desempenho de Papel	Profissional; Enfermeiro; Treinamento; Avaliação; Prescrever; Conhecimento; Educação; Protocolo; Utilizar; Aprender; Etapa; Consulta; Serviço; Clínico; Programa; Implementar; Liderança; Prestador; Planejamento; Iniciativa.
Modo de interdependência	Participante; Participar; Relação; Envolvimento; Consentimento; Confidencialidade; Paciente; Indivíduo; População; Público; Grupo; Diversidade; Atendimento; Acesso; Disponibilidade; Barreira; Necessidade; Apoiar; Fornecer; Oportunidade; Ambiente; Atenção; Sugerir; Representar; Exclusão; Inacessível; Discutir; Intimidade.

Este é um artigo de acesso aberto distribuído sob os termos da Licença de Atribuição Creative Commons.

O modo fisiológico reúne vocábulos relacionados à infecção, transmissão, exposição, profilaxia, exame e monitoramento, expressando os aspectos biológicos e clínicos do cuidado. O modo de autoconceito agrega termos vinculados à percepção, atitude, confiança, disposição e tomada de decisão, refletindo a dimensão subjetiva e psicológica do processo adaptativo. O modo de desempenho de papel concentra palavras associadas à atuação profissional, como enfermeiro, treinamento, avaliação, protocolo, prescrição, serviço e implementação, destacando o papel técnico, educativo e organizacional da enfermagem. Por fim, o modo de interdependência abrange termos relacionados à participação, vínculo, confidencialidade, acesso, apoio, ambiente e relacionamento, evidenciando a importância das interações sociais e institucionais no cuidado.

## DISCUSSÃO

A análise temporal das publicações entre 2018 e 2025 evidencia uma concentração progressiva de estudos a partir de 2018, demonstrando o aumento do interesse científico em torno da PrEP. A configuração observada acompanha tendências já consolidadas na literatura internacional, que assinala a predominância de centros de pesquisa do Norte Global na produção científica sobre PrEP e, simultaneamente, o fortalecimento da participação de países latino-americanos, particularmente o Brasil, nas análises sobre implementação em sistemas públicos de saúde.

A análise lexical evidenciou a construção discursiva do fenômeno da PrEP ao HIV, a partir das percepções, práticas e decisões relacionadas ao cuidado em saúde. O agrupamento realizado por meio do IRaMuTeQ permitiu identificar núcleos de sentido que expressam diferentes eixos conceituais envolvidos na implementação, no acesso e na tomada de decisão acerca da PrEP. Embora a organização das classes decorra do processamento estatístico do software, a inferência clínica fundamentou-se na interpretação dos pesquisadores^(3)^.

As classes lexicais foram padronizadas conforme eixos semânticos que representam: percepção de vulnerabilidade, cuidado profissional, implementação da PrEP e tomada de decisão. Cada classe reúne termos inter-relacionados que, em conjunto, aprofundam a compreensão das barreiras, estratégias e processos que permeiam a prevenção do HIV no contexto dos serviços de saúde, conforme evidenciado no dendrograma e na Análise Fatorial de Correspondência.

A AFC demonstrou que, na Classe 1, os termos evidenciam desafios organizacionais e programáticos, como acesso, disponibilidade e barreiras. Nesse sentido, destaca-se a necessidade de protocolos claros que garantam a oferta da PrEP em diferentes contextos de cuidado, a importância de preparar enfermeiros para lidar com questões estruturais e de gestão de serviços, além da implementação de políticas públicas que assegurem recursos e fluxos assistenciais adequados, ampliando a cobertura e a equidade no acesso^(10,11,12)^.

A Classe 2 concentrou termos ligados a conhecimento, confiança, consentimento e atitude, refletindo processos cognitivos e éticos. Esses achados apontam para a necessidade de o enfermeiro adotar estratégias educativas que promovam escolhas conscientes e adesão à PrEP, compreender os fatores que influenciam a decisão dos usuários quanto ao seu uso e desenvolver campanhas de sensibilização que reduzam barreiras informacionais^(13,14,15)^.

Na Classe 3, os termos relacionados à vulnerabilidade evidenciaram a exigência de práticas centradas no usuário, que considerem sua percepção individual de risco e promovam vínculos de confiança. Ressalta-se, ainda, a importância da formação de enfermeiros com competências em comunicação e abordagem culturalmente sensível. Ao compreender as percepções de vulnerabilidade dos usuários, o enfermeiro poderá orientar estratégias de prevenção mais direcionadas a grupos específicos, ampliando a efetividade das políticas de saúde^(16,17)^.

A Classe 4 reuniu termos que evidenciaram o núcleo técnico-assistencial da prática do enfermeiro. Esse achado reforça o papel do profissional como protagonista na condução da PrEP, desde a prescrição até o acompanhamento clínico. Evidencia, também, a necessidade de capacitação contínua em protocolos e práticas baseadas em evidências, bem como a padronização terminológica como ferramenta para fortalecer indicadores assistenciais, avaliar a qualidade do cuidado e subsidiar decisões estratégicas^(18,19)^.

No que concerne à conexão entre as classes, observou-se proximidade entre a Classe 1 e a Classe 4, evidenciada pela concentração de termos relacionados aos serviços, programas, atuação clínica e práticas profissionais. Essa associação indica que a efetividade da PrEP está diretamente vinculada à capacidade organizacional dos serviços de saúde, ao planejamento das ações e à atuação qualificada dos profissionais, especialmente do enfermeiro, na condução do cuidado preventivo ao HIV^(20,21,22,23)^. Já a conexão entre a Classe 3 e a Classe 2 revela que a proximidade entre termos relacionados à exposição, comportamento e prevenção, com vocábulos associados à atitude, confiança, conhecimento e decisão^(24,25)^, evidencia que a escolha pelo uso da PrEP ultrapassa a dimensão técnica, envolvendo processos subjetivos, cognitivos e emocionais dos indivíduos.

A utilização do Referencial Teórico de Roy possibilitou evidenciar como cada modo traduz dimensões específicas do cuidado de enfermagem relacionadas à PrEP para HIV, permitindo compreender tanto os aspectos clínicos e biológicos quanto os subjetivos, profissionais e relacionais envolvidos no processo assistencial. Assim, a associação entre uma teoria de enfermagem e a análise lexical ampliou a aplicabilidade prática dos resultados, oferecendo subsídios para a assistência, o ensino e a gestão em enfermagem, ao alinhar intervenções técnicas às necessidades humanas e sociais dos usuários.

Os termos relacionados ao modo fisiológico, como infecção, transmissão, exame, monitoramento e profilaxia, evidenciam a dimensão biológica e clínica do cuidado de enfermagem na PrEP. Na assistência, isso implica protocolos mais precisos de rastreamento e acompanhamento clínico, garantindo que os usuários recebam monitoramento contínuo e seguro^(26,27)^. No ensino, reforça a necessidade de capacitar enfermeiros para interpretar exames laboratoriais e orientar sobre medidas preventivas. Já na gestão, a padronização desses registros fortalece indicadores epidemiológicos e permite melhor planejamento de recursos voltados à prevenção do HIV.

O modo de autoconceito, representado por termos como atitude, confiança, percepção e tomada de decisão, revela a dimensão subjetiva e psicológica do cuidado. Na assistência, enfermeiros devem desenvolver estratégias de comunicação que promovam confiança e autonomia dos usuários na escolha pela PrEP. No ensino, os achados apontam para a importância de incluir conteúdos sobre aconselhamento, empoderamento e tomada de decisão nos currículos de enfermagem. Na gestão, compreender esses aspectos subjetivos auxilia na formulação de políticas que valorizem a adesão e reduzam barreiras emocionais ou culturais ao uso da PrEP.

Os termos vinculados ao desempenho de papel, como enfermeiro, treinamento, prescrição, protocolo e implementação, destacam a atuação profissional e organizacional da enfermagem. Na assistência, isso^(28)^ reforça o papel do enfermeiro como protagonista na implementação da PrEP, desde a prescrição até o acompanhamento clínico. No ensino, evidencia a necessidade de preparar profissionais para liderar programas de prevenção, com ênfase em protocolos e práticas baseadas em evidências. Na gestão, a clareza terminológica fortalece a construção de fluxos assistenciais e a definição de responsabilidades, garantindo maior eficiência nos serviços de saúde.

O modo de interdependência, expresso por termos como vínculo, consentimento, confidencialidade, acesso e apoio, evidencia a dimensão relacional e social do cuidado. Na assistência, isso se traduz em práticas que valorizam o vínculo terapêutico e a confidencialidade, fundamentais para populações vulneráveis^(29,30)^. No ensino, os resultados reforçam a necessidade de formar enfermeiros com competências em comunicação, diversidade cultural e ética. Na gestão, a padronização desses aspectos favorece políticas inclusivas, amplia o acesso e fortalece redes de apoio, garantindo que a PrEP seja implementada de forma equitativa e centrada nas necessidades dos usuários.

Essa integração possibilita alinhar intervenções técnicas às dimensões sociais e humanas do cuidado, oferecendo subsídios para o raciocínio clínico do enfermeiro e para o desenvolvimento de intervenções mais efetivas, equitativas e centradas nas necessidades das populações em uso ou potencialmente elegíveis para a PrEP.

A limitação do estudo diz respeito ao uso exclusivo do software IRaMuTeQ, que, embora robusto, depende da consistência do corpus textual e da interpretação dos pesquisadores para a categorização dos termos. Essa característica pode restringir a generalização dos resultados, uma vez que a análise lexical está condicionada à qualidade e ao contexto dos textos incluídos.

Apesar dessas limitações, os resultados desta análise lexical apresentam implicações práticas diretas para a enfermagem em diferentes dimensões. Na assistência, a identificação de termos recorrentes pode orientar a construção de protocolos de registro clínico mais padronizados, favorecendo o uso da CIPE® e garantindo maior precisão na documentação do cuidado. Isso fortalece a segurança do paciente e assegura a continuidade das ações de saúde.

No ensino, os achados indicam conteúdos prioritários para a formação profissional, como tomada de decisão, percepção de vulnerabilidade e implementação da PrEP. Esses elementos podem ser incorporados em currículos de graduação e programas de capacitação, preparando enfermeiros para lidar com cenários complexos de prevenção do HIV e ampliando sua competência clínica e educativa.

Na gestão, a padronização terminológica contribui para a qualificação dos indicadores assistenciais, para o monitoramento de resultados e para o fortalecimento das políticas públicas de saúde. Essa prática permite que gestores utilizem dados mais consistentes na formulação de estratégias de prevenção e na alocação de recursos. Dessa forma, o estudo oferece subsídios concretos para aprimorar a prática clínica, a formação acadêmica e a gestão em enfermagem, ampliando o impacto da atuação profissional na prevenção do HIV.

## CONCLUSÃO

Este estudo evidenciou a natureza multidimensional do cuidado de enfermagem no contexto da PrEP para HIV, ao revelar classes lexicais que articulam dimensões organizacionais, subjetivas, profissionais e relacionais. Os resultados contribuem para fortalecer o raciocínio clínico do enfermeiro, apoiar a padronização dos registros e ampliar a segurança e a qualidade da assistência.

De forma prática, os achados oferecem subsídios para a atuação profissional em diferentes níveis: orientam registros clínicos mais consistentes, apoiam processos formativos e subsidiam políticas de gestão em saúde. A clareza terminológica identificada favorece a comunicação multiprofissional e reforça o papel estratégico da enfermagem na prevenção do HIV.

Como perspectivas futuras, destaca-se a necessidade de avançar na padronização terminológica vinculada à CIPE®, com o desenvolvimento de subconjuntos específicos para a PrEP e sua validação clínica, garantindo maior aplicabilidade dos termos na prática assistencial. Além disso, recomenda-se investir em programas de capacitação de enfermeiros para o uso consistente da linguagem padronizada, ampliando a efetividade das estratégias de prevenção e consolidando práticas baseadas em evidências.

## Data Availability

Todo o conjunto de dados que dá suporte aos resultados deste estudo está disponível mediante solicitação ao autor correspondente.

# Translation: Terminología especializada de enfermería en la profilaxis previa a laexposición: análisis léxico

**DOI:** 10.1590/1980-220X-REEUSP-2026-0166es

**Authors:** José Leonildo Fernandes de Queiroz, Daniela Dias Quirino, Semíramis Mesquita Ciríaco da Silva, Adelson de Oliveira da Cruz, Isaque Augusto Rosendo Costa, Richardson Augusto Rosendo da Silva

**Affiliations:** 1 Universidade Federal do Rio Grande do Norte, Departamento de Enfermagem, Natal, RN, Brazil.; 2 Universidade Federal da Paraíba, Departamento de Enfermagem, João Pessoa, PB, Brazil.; 3 Universidade Federal do Rio Grande do Norte, Departamento de Psicologia, Natal, RN, Brazil.

**Keywords:** Atención de Enfermería, Prevención de Enfermedades, Salud Sexual, VIH, Profilaxis Preexposición

## Abstract

**Objetivo:**

Analizar el léxico de los términos relacionados con la atención de enfermería dirigida a los usuarios de la profilaxis preexposición (PrEP) para el VIH, basándose en la literatura científica.

**Método:**

Revisión de alcance realizada según las directrices de PRISMA-ScR. La identificación de los estudios se llevó a cabo en bases de datos multidisciplinarias y específicas del área de la salud, en repositorios de literatura gris nacionales e internacionales, así como en documentos oficiales del Ministerio de Salud, sin restricciones de idioma o año. Los estudios se sometieron a un análisis léxico mediante el software IRaMuTeQ, y los hallazgos se interpretaron a la luz de la Teoría de la Adaptación de Roy, que orientó la organización de las clases léxicas según los modos fisiológico, autoconcepto, desempeño de roles e interdependencia, lo que permitió comprender el fenómeno de manera multidimensional.

**Resultados:**

Se identificaron 24 245 registros, de los cuales 18 conformaron el corpus final, con un total de 1770 segmentos de texto. Surgieron cuatro clases léxicas: Implementación de la PrEP para el VIH, Cuidado profesional, Percepción de vulnerabilidad y Toma de decisiones.

**Conclusión:**

Se evidenció un vocabulario representativo de la atención en la PrEP, destacando la contribución de la Teoría de Roy para la interpretación de los hallazgos y para la comprensión integrada de las dimensiones de la atención de enfermería.

## INTRODUCCIÓN

La epidemia del virus de la inmunodeficiencia humana (VIH) sigue siendo un desafío global de salud pública, que exige estrategias innovadoras de prevención. En este contexto, la profilaxis preexposición (PrEP), basada en el uso de medicamentos antirretrovirales, se ha consolidado como una herramienta eficaz para reducir la transmisión del virus y ampliar la protección en poblaciones vulnerables^(1)^.

El Proceso de Enfermería (PE) es esencial para sistematizar la atención y sustentar el ejercicio profesional del enfermero^(2)^. Para ello, es necesario calificar continuamente la práctica, sobre todo mediante el uso de Sistemas de Lenguaje Estandarizado (Sistema de Linguagem Padronizado -SLP), que favorecen la comunicación multiprofesional y la continuidad de las acciones de salud. Entre los SLP, destaca la Clasificación Internacional para la Práctica de Enfermería (CIPE®), reconocida por su amplitud y capacidad para respaldar registros clínicos precisos^(3,4)^.

Sin embargo, se observa que, en la práctica clínica, persisten lagunas en el uso y el registro de los términos relacionados con la atención de enfermería en el contexto de la PrEP para el VIH. Esta fragilidad compromete la sistematización de las acciones, la estandarización del lenguaje y la eficacia de la comunicación entre los profesionales de la salud^(5)^.

El análisis léxico de la literatura científica surge, en este sentido, como una estrategia prometedora para identificar los conceptos más recurrentes sobre el tema, revelando creencias, prácticas y dimensiones de la atención a la luz de los discursos académicos de la enfermería.

La lexicografía proporciona una base teórica y metodológica para comprender el lenguaje en uso, permitiendo no solo la identificación de términos relevantes, sino también la organización de núcleos de sentido en clases temáticas representativas de la práctica cotidiana^(6)^.

En enfermería, el análisis léxico contribuye a comprender fenómenos complejos^(7)^, como la atención a los usuarios de la PrEP. Favorece la construcción de subconjuntos terminológicos de CIPE^®^ más consistentes, fortalece el sistema clasificatorio, amplía la estandarización del lenguaje y mejora la calidad de los registros clínicos, lo que justifica la realización del presente estudio.

La relevancia del estudio reside no solo en la innovación del enfoque, sino también en su capacidad para respaldar políticas de salud y prácticas profesionales más calificadas, favoreciendo la comunicación multiprofesional y el registro clínico preciso. Se trata de una producción que contribuye a consolidar el papel de la enfermería en la prevención del VIH y en la implementación de la PrEP, con impacto directo en la calidad de la atención y en la seguridad de los usuarios.

Ante esto, se plantea la pregunta: ¿cómo se organizan léxicamente en la literatura científica los términos relacionados con la atención de enfermería a los usuarios de la PrEP para el VIH? Así, este estudio tiene como objetivo analizar el léxico de los términos referentes a la atención de enfermería dirigida a los usuarios de la PrEP para el VIH, basándose en la literatura científica.

## MÉTODO

### Diseño del Estudio

Se trata de una *scoping review*, guiada por un protocolo de investigación previamente registrado en el Open Science Framework (https://doi.org/10.17605/OSF.IO/N3PMX). El estudio se llevó a cabo de acuerdo con las recomendaciones del *Joanna Briggs Institute Reviewer’s Manual* y las directrices PRISMA-ScR^(8)^.

La pregunta orientadora se construyó mediante la estrategia PCC, definida como: P (Población) Usuarios de la PrEP para el VIH; C (Concepto) Términos relacionados con la atención de enfermería; y C (Contexto) Literatura científica sobre la PrEP y la atención de enfermería. Así, se estableció la siguiente pregunta: ¿Qué términos relacionados con la atención de enfermería dirigidos a los usuarios de la PrEP para el VIH se identifican y describen en la literatura científica?

### Lugar del Estudio

Las búsquedas se realizaron en bases de datos electrónicas y repositorios de literatura gris, utilizando el Portal de Periódicos de CAPES, a través de la Comunidad Académica Federal (CAFe).

### Población y Criterios de Selección

Se incluyeron artículos alineados con el objetivo de la investigación, publicados en bases indexadas nacionales e internacionales, sin restricciones temporales ni de idioma, además de literatura gris (tesis, disertaciones y documentos técnicos) y, como criterios de exclusión, estudios sin resultados completos o solo notas preliminares.

### Recolección de Datos

La identificación de los estudios se realizó en bases de datos electrónicas multidisciplinarias y literatura gris: Scopus; Web of Science; PubMed®; Cumulative Index to Nursing and Allied Health Literature (CINAHL); Cochrane Library; Literatura Latino-Americana e do Caribe em Ciências da Saúde (LiLaCS); SciELO; Embase^®^; Education Resources Information Center (ERIC); Google Acadêmico; DART-Europe; E-Theses Portal; Electronic Theses Online Service (EthOS); Repositório Científico de Acesso Aberto de Portugal (RCAAP); Global ETD Search; Open Access Theses and Dissertations (OATD); Theses Canada; Portal de Tesis Latinoamericanas; World Cat Dissertations and Theses (OCLC); Catálogo de Teses e Dissertações (CAPES); Biblioteca Digital Brasileira de Teses e Dissertações (BDTD); World Wide Science; Protocolos del Ministerio de Salud de Brasil; Notas Técnicas del Ministerio de Salud de Brasil; Dictámenes del Consejo Federal de Enfermería y de los Consejos Regionales de Enfermería de Brasil, así como una búsqueda manual en las listas de referencias de los estudios incluidos, con el fin de identificar pruebas que no se hubieran detectado anteriormente.

Las búsquedas se realizaron en noviembre de 2025 y se estructuraron con descriptores indexados (MeSH/DeCS) y términos libres, combinados con los operadores booleanos AND y OR, adaptados a cada base de datos. Palabras clave principales: *Nursing, Pre-Exposure Prophylaxis, Anti-HIV Agents, Disease Prevention, Sexual Health, Nurse’s Role, Practice Patterns, Nurse’s.* Pare ello fueron realizados los siguiente cruzamientos: ((“Nursing” or “Nurse’s Role” or “Practice Patterns, Nurses’”) and (“Pre-Exposure Prophylaxis” or “Disease Prevention” or “Anti-HIV Agents”) and (“Sexual Health”)).

La selección de los estudios se llevó a cabo en tres etapas: cribado, lectura completa y consenso. Para ello, se utilizó el software Rayyan - Intelligent Systematic Review (https://rayyan.ai/). Dos investigadores analizaron, de forma independiente, los títulos y los resúmenes; los estudios elegibles se leyeron en su totalidad. Las discrepancias fueron resueltas por un tercer investigador, y los estudios duplicados se consideraron solo una vez.

La extracción de datos se realizó mediante un instrumento estructurado en Microsoft Excel 2019, llevado a cabo por los dos investigadores, y las discrepancias se resolvieron por consenso. El instrumento incluyó la identificación del estudio (país, año, tipo de publicación, tipo de estudio y nivel de evidencia).

### Análisis y Tratamiento de los Datos

Los estudios se clasificaron según el nivel de evidencia del Oxford Centre for Evidence-Based Medicine (OCEBM), de acuerdo con la clasificación del JBI (niveles 1 a 4). Las evidencias variaron desde el nivel 1 (síntesis sólidas) hasta el nivel 4 (estudios descriptivos e informes de experiencia).

El corpus textual, constituido a partir de resúmenes, resultados y consideraciones finales de los estudios seleccionados, se sometió al software IRaMuTeQ. Este programa aplicó técnicas estadísticas de análisis léxico, como la CJD y el Análisis Factorial de Correspondencia (AFC), que permiten segmentar el texto en unidades de contexto y agrupar los términos por similitud semántica y frecuencia de ocurrencia. La CJD permitió identificar clases léxicas homogéneas y semánticamente coherentes, mientras que la AFC puso de manifiesto la distribución espacial y relacional de estas clases, lo que facilitó la visualización de las conexiones entre los núcleos de sentido. Este proceso garantizó el rigor metodológico y sustentó la interpretación de los hallazgos a la luz de la Teoría de la Adaptación de Roy, que orientó la organización de las clases en dimensiones fisiológicas, subjetivas, profesionales y relacionales de la atención de enfermería.

Los resultados se presentaron mediante el diagrama de flujo PRISMA-ScR (identificación, selección, elegibilidad e inclusión), un cuadro con características metodológicas y hallazgos principales, cuadros con un resumen del nivel de evidencia y estrategias de búsqueda, y figuras del IRaMuTeQ (dendrograma de la CJD y mapa factorial del AFC).

La elección del análisis léxico mediante el software IRaMuTeQ se justificó por su pertinencia en la identificación de núcleos de sentido y patrones de uso del lenguaje en textos científicos. En el ámbito de la construcción de terminologías de la CIPE®, este enfoque representa una etapa inicial de mapeo y organización de los términos, proporcionando bases para la elaboración de subconjuntos terminológicos más consistentes. Se trata, por lo tanto, de una estrategia complementaria que precede a los procesos de validación clínica, contribuyendo a fortalecer la estandarización del lenguaje y la aplicabilidad práctica de la CIPE® en los registros de enfermería.

Además, la elección de la Teoría de la Adaptación de Callista Roy como marco analítico del estudio se basó en su capacidad para interpretar fenómenos complejos de la atención de enfermería a partir de cuatro modos adaptativos: fisiológico, autoconcepto, desempeño de roles e interdependencia. Esta estructura teórica resultó adecuada para el objetivo del análisis terminológico, ya que^(9)^ permitió organizar los hallazgos léxicos en dimensiones que reflejan tanto aspectos clínicos y biológicos como subjetivos, relacionales y organizacionales de la atención.

### Aspectos Éticos

Al tratarse de una revisión bibliográfica basada en datos secundarios de acceso público, no fue necesario someterla al Comité de Ética en Investigación. Cabe destacar, sin embargo, que se respetaron los principios éticos relacionados con los derechos de autor y la cita adecuada de las fuentes.

## RESULTADOS

El proceso de revisión se inició con la identificación de 24247 registros en diferentes bases de datos y mediante búsqueda manual. En la etapa de selección, se excluyeron 24223 registros por no cumplir con los criterios de elegibilidad, por duplicidad o irrecuperabilidad, por irrelevancia temática o por no responder a la pregunta central. Paralelamente, de los cuatro informes/manuales analizados mediante métodos complementarios, se mantuvieron todos. Al final del proceso de selección, se incluyeron 18 estudios en la muestra final de esta revisión (Figura 1).

**Figura 1:** Diagrama de flujo para la revisión exploratoria (adaptado de Prisma). Natal, RN, Brasil, 2025.

El corpus textual final de la investigación estuvo compuesto por 18 documentos, presentados en la Cuadro 1, los cuales fueron procesados y segmentados en 1770 segmentos de texto (ST), 1566 (88,47%) de los cuales fueron clasificados mediante el análisis léxico. Tras el análisis de los datos, estos fueron organizados y presentados en la tabla. Para facilitar la comprensión y la visualización de los resultados, los estudios se identificaron con la letra «E» (estudio), seguida de números arábigos secuenciales (1, 2, 3,...18), lo que dio lugar a las codificaciones E1, E2, E3,... E18 (Cuadro 1).

**Cuadro 1:** Caracterización de los estudios seleccionados para la investigación – Natal, RN, Brasil, 2025.

Estudio	País (año)	Tipo de publicación/ Tipo de estudio / Nivel de evidencia
E1^(6)^	Australia (2018)	Artículo científico / Estudio clínico / 3
E2^(7)^	Canadá (2018)	Artículo científico / Estudio observacional correlacional / 4
E3^(8)^	Estados Unidos (2019)	Artículo científico / Estudio metodológico / 4
E4^(9)^	Estados Unidos (2019)	Artículo científico / Estudio observacional correlacional / 4
E5^(10)^	Canadá (2020)	Artículo científico / Estudio observacional / 4
E6^(11)^	Canadá (2020)	Artículo científico / Estudio observacional retrospectivo / 4
E7^(12)^	Canadá (2020)	Artículo científico / Estudio observacional longitudinal / 4
E8^(13)^	Brasil (2020)	Dictamen técnico / Documento institucional / NE^*^
E9^(14)^	Canadá (2021)	Artículo científico / Estudio observacional / 4
E10^(15)^	Canadá (2022)	Artículo científico / Estudio cualitativo / 5
E11^(16)^	Estados Unidos (2022)	Artículo científico / Protocolo de ensayo clínico / 3
E12^(17)^	Brasil (2022)	Directriz/Protocolo / Síntesis de la evidencia / NE^*^
E13^(18)^	Canadá (2023)	Artículo científico / Estudio cualitativo / 4
E14^(19)^	Canadá (2023)	Artículo científico / Estudio observacional / 4
E15^(20)^	Brasil (2023)	Nota técnica / Documento institucional / NE^*^
E16^(16)^	Brasil (2024)	Nota técnica / Documento institucional / NE^*^
E17^(17)^	Estados Unidos (2025)	Artículo científico / Estudio cualitativo / 4
E18^(18)^	Australia (2025)	Artículo científico / Estudio observacional / 3

*NE (no aptos para la clasificación OCEBM).

El análisis realizado con el software IRaMuTeQ permitió clasificar los segmentos de 18 textos en cuatro clases temáticas distintas. Con el fin de garantizar la uniformidad terminológica, las clases se denominaron en singular y se estandarizaron de la siguiente manera: Clase 1 - Implementación de la PrEP (27,2%); Clase 2: Toma de decisiones (23,3%); Clase 3: Percepción de vulnerabilidad (14%); Clase 4: Atención profesional (35,5%), tal y como se muestra en la (Figura 2).

**Figura 2:** Diagrama de las clases que componen el dendograma del corpus textual correspondiente a los manuscritos de la muestra. Natal, RN, Brasil, 2025.

Las clases léxicas identificadas no se presentan de forma aislada, sino que se articulan como etapas interdependientes del proceso de atención relacionado con la PrEP. Este recorrido se inicia con la implementación de la PrEP en los servicios de salud (Clase 1) y con la toma de decisiones de los usuarios (Clase 2), que expresan dimensiones organizativas, programáticas y cognitivas de la atención. A continuación, surgen las clases relacionadas con la percepción de la vulnerabilidad (Clase 3) y la atención profesional (Clase 4), que incorporan aspectos subjetivos, relacionales y asistenciales, incluyendo el papel del profesional de la salud, especialmente del personal de enfermería, en la prevención y el seguimiento.

La relación entre las clases debe analizarse de izquierda a derecha, siguiendo la lógica de la Clasificación Jerárquica Descendente. En el presente estudio, el corpus se sometió a una única división, de la que resultaron cuatro subcorpus, correspondientes a las Clases 3 (Percepción de la Vulnerabilidad), 4 (Cuidado Profesional), 1 (Implementación de la PrEP) y 2 (Toma de Decisiones). Esta configuración indica que las clases surgieron simultáneamente a partir del mismo proceso de segmentación, sin subdivisiones sucesivas del corpus. La interrupción de la clasificación se produjo debido a la estabilidad alcanzada, evidenciada por la constitución de conjuntos de segmentos de texto con un vocabulario internamente homogéneo y semánticamente coherente, lo que permitió el análisis integrado de las clases como dimensiones complementarias del fenómeno investigado (Figura 2).

El análisis factorial de correspondencias (AFC) permitió mapear la distribución espacial de estas clases (Figura 3). Las clases identificadas se relacionan directamente con la práctica de la atención de salud y la enfermería en el contexto de la prevención del VIH y la implementación de la PrEP, ya que reflejan dimensiones esenciales del proceso asistencial.

**Figura 3:** Análisis factorial de correspondencias de las palabras más frecuentes en cada clase léxica, basado en una clasificación jerárquica. Natal, RN, Brasil, 2025.

Los hallazgos léxicos relacionados con la implementación de la PrEP y la toma de decisiones (Clases 1 y 2) influyen en la organización de los servicios, la disponibilidad de recursos y las condiciones de acceso, al tiempo que se articulan con los procesos formativos, informativos y éticos que sustentan las elecciones conscientes y la adhesión a las estrategias preventivas. A su vez, las clases referidas a la percepción de vulnerabilidad y a la atención profesional (Clases 3 y 4) refuerzan la importancia de comprender la vulnerabilidad individual, el comportamiento preventivo y el vínculo establecido entre los usuarios y los profesionales de la salud.

El análisis factorial de correspondencias puso de manifiesto la organización semántica y léxica de los términos más frecuentes en el corpus, lo que permitió visualizar la estructura relacional entre las clases identificadas. Tal como se ilustra en el plano factorial, la Clase 2 —Toma de decisiones (representada en el cuadrante superior izquierdo)— concentra términos asociados a capacitación, conocimiento, educación, evaluación, confianza, consentimiento y actitud, lo que indica una fuerte articulación entre procesos cognitivos, informacionales y decisorios. La posición de esta clase sugiere la centralidad de los aspectos formativos y de la autonomía de los sujetos en el contexto analizado.

En el cuadrante inferior izquierdo, se observa la Clase 1 - Implementación de la PrEP, caracterizada por términos como servicio, programa, implementar, acceso, disponibilidad, población, barrera y prevención. La proximidad espacial entre las Clases 1 y 2 indica una elevada correlación semántica, revelando que los procesos de toma de decisiones están intrínsecamente relacionados con las condiciones organizativas y programáticas de los servicios de salud responsables de la oferta de la PrEP.

En el cuadrante superior derecho, destaca la Clase 3 - Percepción de Vulnerabilidad, compuesta por términos como sexo, comportamiento, exposición, preservativo y transmisión. Esta clase expresa la dimensión subjetiva y conductual de la atención, relacionada con la interpretación individual de la vulnerabilidad y las prácticas de prevención. Su posición en el plano factorial indica una relativa autonomía conceptual, aunque mantiene una interfaz con los demás dominios de la atención.

Por último, en el cuadrante inferior derecho se concentra la Clase 4 - Cuidado profesional, fuertemente asociada a términos como enfermero, profesional, salud, infección, transmisible, monitoreo y examen. El relativo aislamiento de esta clase en el plano factorial pone de manifiesto la especificidad del campo léxico vinculado a la práctica clínica y al saber técnico-profesional, lo que indica que, aunque está relacionada con las demás clases, esta dimensión se estructura a partir de un núcleo conceptual propio.

En conjunto, la distribución de las clases en el plano factorial revela la complejidad del discurso analizado, al explicitar cómo las dimensiones organizativas, decisorias, subjetivas y técnico- asistenciales se articulan en el contexto de la prevención del VIH y la implementación de la PrEP. El AFC permite, así, una lectura integrada de los patrones de asociación entre términos y clases, reforzando la naturaleza multidimensional de la atención en salud y de la práctica de la enfermería.

En el Cuadro 2, los términos extraídos del corpus se organizaron según los cuatro modos adaptativos de la Teoría de la Adaptación de Roy, evidenciando la naturaleza multidimensional del fenómeno analizado en el contexto de la prevención del VIH y la implementación de la PrEP.

**Cuadro 2:** Términos relacionados con la atención en el contexto de la PrEP para el VIH a la luz del marco teórico de la Adaptación de Callista Roy – Natal, RN, Brasil, 2025.

Marco teórico de adaptación de Roy	
Modos adaptativos	Términos relacionados
Modo fisiológico	Transmisible; Infección; Salud; Preservativo; Prueba; Seguimiento; Profilaxis; Exposición; Alto; Contraer; Adquisición; Adquirir; Inyectar; Tomar; Transmisión; Detectable; Negativo; Prevenir; Probabilidad; Índice.
Modo de autoconcepto	Actitud; Confianza; Disposición; Concienciación; Percepción; Percibir; Cómodo; Preocuparse; Beneficio; Decidir; Deber; Convertir.
Modo de desempeño del rol	Profesional; Enfermero; Formación; Evaluación; Prescribir; Conocimiento; Educación; Protocolo; Utilizar; Aprender; Etapa; Consulta; Servicio; Clínico; Programa; Implementar; Liderazgo; Prestador; Planificación; Iniciativa.
Modo de interdependencia	Participante; Participar; Relación; Implicación; Consentimiento; Confidencialidad; Paciente; Individuo; Población; Público; Grupo; Diversidad; Atención; Acceso; Disponibilidad; Barrera; Necesidad; Apoyar; Proporcionar; Oportunidad; Entorno; Cuidado; Sugerir; Representar; Exclusión; Inaccesible; Debatir; Intimidad.

Este es un artículo de acceso abierto, distribuído bajo los términos de la Licencia Creative Commons.

El modo fisiológico reúne vocabulario relacionado con la infección, la transmisión, la exposición, la profilaxis, el examen y el monitoreo, expresando los aspectos biológicos y clínicos de la atención. El modo de autoconcepto agrega términos vinculados a la percepción, la actitud, la confianza, la disposición y la toma de decisiones, reflejando la dimensión subjetiva y psicológica del proceso adaptativo. El modo de desempeño de roles concentra palabras asociadas a la actuación profesional, como enfermero, capacitación, evaluación, protocolo, prescripción, servicio e implementación, destacando el papel técnico, educativo y organizacional de la enfermería. Por último, el modo de interdependencia abarca términos relacionados con la participación, el vínculo, la confidencialidad, el acceso, el apoyo, el entorno y las relaciones, evidenciando la importancia de las interacciones sociales e institucionales en la atención.

## DISCUSIÓN

El análisis temporal de las publicaciones entre 2018 y 2025 pone de manifiesto una concentración progresiva de estudios a partir de 2018, lo que demuestra el creciente interés científico en torno a la PrEP. La configuración observada sigue tendencias ya consolidadas en la literatura internacional, que señala el predominio de los centros de investigación del Norte Global en la producción científica sobre la PrEP y, al mismo tiempo, el fortalecimiento de la participación de los países latinoamericanos, particularmente Brasil, en los análisis sobre la implementación en los sistemas de salud pública.

El análisis léxico evidenció la construcción discursiva del fenómeno de la PrEP frente al VIH, a partir de las percepciones, prácticas y decisiones relacionadas con la atención en salud. La agrupación realizada mediante el IRaMuTeQ permitió identificar núcleos de sentido que expresan diferentes ejes conceptuales involucrados en la implementación, el acceso y la toma de decisiones sobre la PrEP. Aunque la organización de las clases se deriva del procesamiento estadístico del software, la inferencia clínica se basó en la interpretación de los investigadores^(3)^.

Las clases léxicas se estandarizaron según ejes semánticos que representan: percepción de vulnerabilidad, atención profesional, implementación de la PrEP y toma de decisiones. Cada clase reúne términos interrelacionados que, en conjunto, profundizan la comprensión de las barreras, estrategias y procesos que impregnan la prevención del VIH en el contexto de los servicios de salud, tal como se evidencia en el dendrograma y en el Análisis Factorial de Correspondencia.

El AFC demostró que, en la Clase 1, los términos evidencian desafíos organizacionales y programáticos, como el acceso, la disponibilidad y las barreras. En este sentido, se destaca la necesidad de protocolos claros que garanticen la oferta de la PrEP en diferentes contextos de atención, la importancia de preparar a los enfermeros para lidiar con cuestiones estructurales y de gestión de servicios, además de la implementación de políticas públicas que aseguren recursos y flujos asistenciales adecuados, ampliando la cobertura y la equidad en el acceso^(10,11,12)^.

La Clase 2 agrupó términos relacionados con el conocimiento, la confianza, el consentimiento y la actitud, reflejando procesos cognitivos y éticos. Estos hallazgos apuntan a la necesidad de que el personal de enfermería adopte estrategias educativas que promuevan elecciones conscientes y la adherencia a la PrEP, comprenda los factores que influyen en la decisión de los usuarios respecto a su uso y desarrolle campañas de sensibilización que reduzcan las barreras informativas^(13,14,15)^.

En la Clase 3, los términos relacionados con la vulnerabilidad evidenciaron la exigencia de prácticas centradas en el usuario, que consideren su percepción individual del riesgo y promuevan vínculos de confianza. Cabe destacar, además, la importancia de la formación de enfermeros con competencias en comunicación y un enfoque culturalmente sensible. Al comprender las percepciones de vulnerabilidad de los usuarios, el enfermero podrá orientar estrategias de prevención más dirigidas a grupos específicos, ampliando la eficacia de las políticas de salud^(16,17)^.

La Clase 4 reunió términos que evidenciaron el núcleo técnico-asistencial de la práctica del enfermero. Este hallazgo refuerza el papel del profesional como protagonista en la gestión de la PrEP, desde la prescripción hasta el seguimiento clínico. Evidencia, además, la necesidad de capacitación continua en protocolos y prácticas basadas en la evidencia, así como la estandarización terminológica como herramienta para fortalecer los indicadores asistenciales, evaluar la calidad de la atención y respaldar las decisiones estratégicas^(18,19)^.

En cuanto a la conexión entre las clases, se observó una proximidad entre la Clase 1 y la Clase 4, evidenciada por la concentración de términos relacionados con los servicios, los programas, la actuación clínica y las prácticas profesionales. Esta asociación indica que la efectividad de la PrEP está directamente vinculada a la capacidad organizacional de los servicios de salud, a la planificación de las acciones y a la actuación calificada de los profesionales, especialmente del personal de enfermería, en la gestión de la atención preventiva del VIH^(20,21,23)^. Por su parte, la conexión entre la Clase 3 y la Clase 2 revela que la proximidad entre términos relacionados con la exposición, el comportamiento y la prevención, con vocabulario asociado a la actitud, la confianza, el conocimiento y la decisión^(24,25)^, evidencia que la elección del uso de la PrEP trasciende la dimensión técnica, involucrando procesos subjetivos, cognitivos y emocionales de los individuos.

El uso del Referencial Teórico de Roy permitió evidenciar cómo cada modo traduce dimensiones específicas de la atención de enfermería relacionadas con la PrEP para el VIH, lo que permitió comprender tanto los aspectos clínicos y biológicos como los subjetivos, profesionales y relacionales involucrados en el proceso asistencial. Así, la asociación entre una teoría de enfermería y el análisis léxico amplió la aplicabilidad práctica de los resultados, ofreciendo aportaciones para la atención, la enseñanza y la gestión en enfermería, al alinear las intervenciones técnicas con las necesidades humanas y sociales de los usuarios.

Los términos relacionados con el modo fisiológico, como infección, transmisión, examen, monitoreo y profilaxis, evidencian la dimensión biológica y clínica de la atención de enfermería en la PrEP. En la atención, esto implica protocolos más precisos de detección y seguimiento clínico, garantizando que los usuarios reciban un monitoreo continuo y seguro^(26,27)^. En la enseñanza, refuerza la necesidad de capacitar a los enfermeros para interpretar exámenes de laboratorio y orientar sobre medidas preventivas. En cuanto a la gestión, la estandarización de estos registros fortalece los indicadores epidemiológicos y permite una mejor planificación de los recursos destinados a la prevención del VIH.

El modo de autoconcepto, representado por términos como actitud, confianza, percepción y toma de decisiones, revela la dimensión subjetiva y psicológica de la atención. En la asistencia, los enfermeros deben desarrollar estrategias de comunicación que promuevan la confianza y la autonomía de los usuarios en la elección de la PrEP. En la enseñanza, los hallazgos apuntan a la importancia de incluir contenidos sobre asesoramiento, empoderamiento y toma de decisiones en los planes de estudio de enfermería. En la gestión, comprender estos aspectos subjetivos ayuda a formular políticas que valoren la adherencia y reduzcan las barreras emocionales o culturales al uso de la PrEP.

Los términos vinculados al desempeño de un rol, como enfermero, capacitación, prescripción, protocolo e implementación, resaltan la actuación profesional y organizacional de la enfermería. En la atención, esto^(28)^ refuerza el papel del enfermero como protagonista en la implementación de la PrEP, desde la prescripción hasta el seguimiento clínico. En la enseñanza, pone de manifiesto la necesidad de preparar a los profesionales para liderar programas de prevención, con énfasis en protocolos y prácticas basadas en la evidencia. En la gestión, la claridad terminológica fortalece la construcción de flujos asistenciales y la definición de responsabilidades, garantizando una mayor eficiencia en los servicios de salud.

El modo de interdependencia, expresado por términos como vínculo, consentimiento, confidencialidad, acceso y apoyo, pone de manifiesto la dimensión relacional y social de la atención. En la asistencia, esto se traduce en prácticas que valorizan el vínculo terapéutico y la confidencialidad, fundamentales para las poblaciones vulnerables^(29,30)^. En la enseñanza, los resultados refuerzan la necesidad de formar enfermeros con competencias en comunicación, diversidad cultural y ética. En la gestión, la estandarización de estos aspectos favorece políticas inclusivas, amplía el acceso y fortalece las redes de apoyo, garantizando que la PrEP se implemente de manera equitativa y centrada en las necesidades de los usuarios.

Esta integración permite alinear las intervenciones técnicas con las dimensiones sociales y humanas de la atención, ofreciendo apoyo para el razonamiento clínico del enfermero y para el desarrollo de intervenciones más efectivas, equitativas y centradas en las necesidades de las poblaciones que utilizan o son potencialmente elegibles para la PrEP.

La limitación del estudio se refiere al uso exclusivo del software IRaMuTeQ, que, aunque robusto, depende de la consistencia del corpus textual y de la interpretación de los investigadores para la categorización de los términos. Esta característica puede restringir la generalización de los resultados, ya que el análisis léxico está condicionado a la calidad y al contexto de los textos incluidos.

A pesar de estas limitaciones, los resultados de este análisis léxico presentan implicaciones prácticas directas para la enfermería en diferentes dimensiones. En la atención, la identificación de términos recurrentes puede orientar la construcción de protocolos de registro clínico más estandarizados, favoreciendo el uso de la CIPE® y garantizando una mayor precisión en la documentación de la atención. Esto fortalece la seguridad del paciente y asegura la continuidad de las acciones de salud.

En la enseñanza, los hallazgos indican contenidos prioritarios para la formación profesional, como la toma de decisiones, la percepción de la vulnerabilidad y la implementación de la PrEP. Estos elementos pueden incorporarse en los planes de estudio de pregrado y en los programas de capacitación, preparando a los enfermeros para lidiar con escenarios complejos de prevención del VIH y ampliando su competencia clínica y educativa.

En la gestión, la estandarización terminológica contribuye a la calificación de los indicadores de atención, al seguimiento de los resultados y al fortalecimiento de las políticas públicas de salud. Esta práctica permite a los gestores utilizar datos más consistentes en la formulación de estrategias de prevención y en la asignación de recursos. De este modo, el estudio ofrece aportaciones concretas para mejorar la práctica clínica, la formación académica y la gestión en enfermería, ampliando el impacto de la actuación profesional en la prevención del VIH.

## CONCLUSIÓN

Este estudio puso de manifiesto la naturaleza multidimensional de la atención de enfermería en el contexto de la PrEP para el VIH, al revelar clases léxicas que articulan dimensiones organizacionales, subjetivas, profesionales y relacionales. Los resultados contribuyen a fortalecer el razonamiento clínico del enfermero, respaldar la estandarización de los registros y ampliar la seguridad y la calidad de la atención.

En la práctica, los hallazgos ofrecen aportaciones para la actuación profesional en diferentes niveles: orientan registros clínicos más consistentes, respaldan los procesos formativos y sustentan las políticas de gestión en salud. La claridad terminológica identificada favorece la comunicación multiprofesional y refuerza el papel estratégico de la enfermería en la prevención del VIH.

Como perspectivas futuras, se destaca la necesidad de avanzar en la estandarización terminológica vinculada al CIPE®, con el desarrollo de subconjuntos específicos para la PrEP y su validación clínica, garantizando una mayor aplicabilidad de los términos en la práctica asistencial. Además, se recomienda invertir en programas de capacitación de enfermeros para el uso consistente del lenguaje estandarizado, ampliando la efectividad de las estrategias de prevención y consolidando prácticas basadas en la evidencia.

## Data Availability

El conjunto de datos que respaldan las conclusiones de este estudio está disponible previa solicitud al autor correspondiente.
